# 5,11,17,23,29-Penta-*tert*-butyl-31,32,33,34,35-penta­propoxycalix[5]arene dichloro­methane hemisolvate

**DOI:** 10.1107/S1600536811000456

**Published:** 2011-01-08

**Authors:** Michaela Pojarová, Michal Dušek, Jan Budka, Ivana Císařová, Emanuel Makrlík

**Affiliations:** a Institute of Physics, AS CR, v.v.i, Na Slovance 2, 182 21 Prague 8, Czech Republic; b Institute of Chemical Technology, Department of Organic Chemistry, Technická 5, 166 28 Prague 6, Czech Republic; c Charles University in Prague, Faculty of Science - Department of Inorganic Chemistry, Hlavova 2030/8, 128 43 Prague 2, Czech Republic; d West Bohemia University, Faculty of Applied Science, Univerzitní 22, 306 14 Plzeň, Czech Republic

## Abstract

The title compound, *tert*-butyl­propoxycalix[5]arene, C_70_H_100_O_5_·0.5CH_2_Cl_2_, crystallizes as a solvate with two mol­ecules of calix[5]arene in 1,2-alternate conformations and one mol­ecule of dichloro­methane in the asymmetric unit. One *tert*-butyl group in one of the mol­ecules and two in the other are disordered over two positions with occupancy factors fixed at 0.5917:0.4083, 0.5901:0.4099 and 0.8535:0.1465, respectively, in the final refinement. The C atoms of a prop­oxy group in each of the mol­ecules are also disordered over two positions with occupancies of 0.7372:0.2628 and 0.5027:0.4973. The mol­ecules form intra­molecular hydrogen bonds between prop­oxy O atoms and an adjacent CH_2_ group in a neighbouring prop­oxy chain. In the crystal, inter­molecular C—H⋯O and C—H⋯Cl inter­actions occur involving the dichloro­methane mol­ecule.

## Related literature

For the synthesis and NMR analysis of esters of *p*-*tert*-butyl­calix[5]arene, see: Stewart *et al.* (1995 ▶). For the weighting scheme used, see: Watkin (1994 ▶); Prince (1982 ▶).
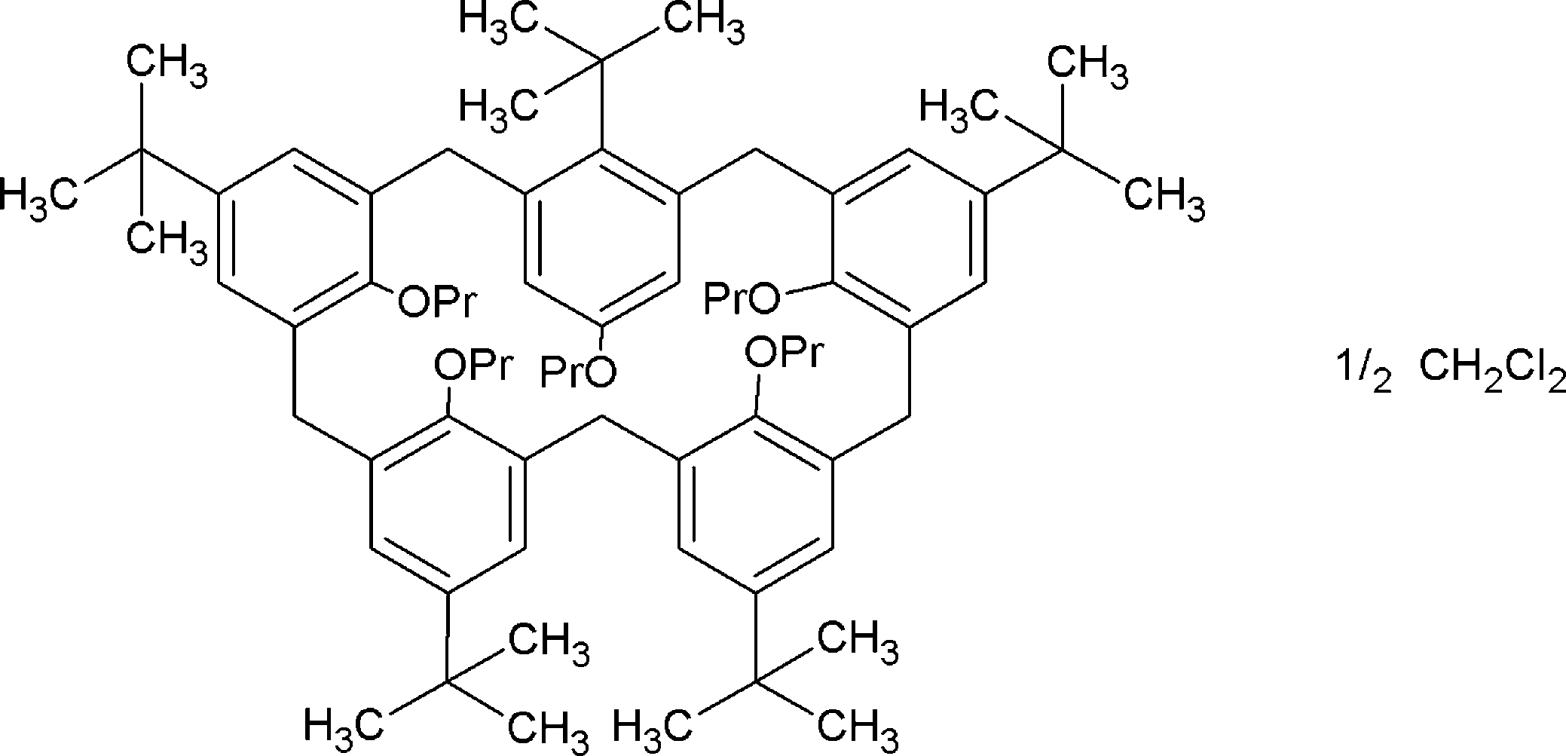


## Experimental

### 

#### Crystal data

C_70_H_100_O_5_·0.5CH_2_Cl_2_
*M* *_r_* = 1064.03
Monoclinic, 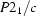

*a* = 12.3740 (2) Å
*b* = 23.9130 (2) Å
*c* = 43.6550 (5) Å
β = 91.7460 (5)°
*V* = 12911.5 (3) Å^3^
*Z* = 8
Mo *K*α radiation
μ = 0.11 mm^−1^
*T* = 150 K
0.45 × 0.23 × 0.12 mm

#### Data collection

Nonius KappaCCD diffractometer
39073 measured reflections
20282 independent reflections
13395 reflections with *I* > 2σ(*I*)
*R*_int_ = 0.043
θ_max_ = 24.1°

#### Refinement

*R*[*F* ^2^ > 2σ(*F* ^2^)] = 0.074
*wR*(*F* ^2^) = 0.151
*S* = 1.02
20282 reflections
1531 parameters
220 restraints
H-atom parameters constrained
Δρ_max_ = 1.88 e Å^−3^
Δρ_min_ = −1.75 e Å^−3^

### 

Data collection: *CrysAlis PRO* (Oxford Diffraction, 2010 ▶); cell refinement: *CrysAlis PRO*; data reduction: *CrysAlis PRO*; program(s) used to solve structure: *SIR97* (Altomare *et al.*, 1999 ▶); program(s) used to refine structure: *CRYSTALS* (Betteridge *et al.*, 2003 ▶); molecular graphics: *DIAMOND* (Brandenburg & Putz, 2005 ▶); software used to prepare material for publication: *CRYSTALS* and *publCIF* (Westrip, 2010 ▶).

## Supplementary Material

Crystal structure: contains datablocks global, I. DOI: 10.1107/S1600536811000456/sj5077sup1.cif

Structure factors: contains datablocks I. DOI: 10.1107/S1600536811000456/sj5077Isup2.hkl

Additional supplementary materials:  crystallographic information; 3D view; checkCIF report

## Figures and Tables

**Table 1:** Hydrogen-bond geometry (Å, °)

*D*—H⋯*A*	*D*—H	H⋯*A*	*D*⋯*A*	*D*—H⋯*A*
C71—H711⋯O7	1.03	2.36	3.363 (8)	163
C48—H482⋯O1	0.99	2.60	3.351 (5)	134
C110—H1101⋯O8	1.00	2.57	3.361 (5)	136
C120—H1202⋯O9	1.00	2.57	3.371 (5)	136
C14—H142⋯O3	1.01	2.47	2.869 (5)	103
C38—H382⋯Cl1	0.97	2.92	3.795 (5)	151

**Fig. 1.**
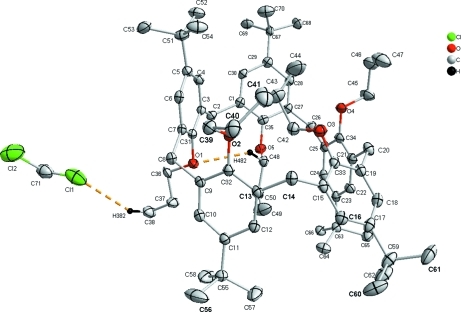
A view of first molecule of 1 with atom's numbering. Displacement ellipsoids are drawn at the 30% probability level. Hydrogen bonds are drawn as dashed lines. Hydrogen atoms not participating in hydrogen bonds are omitted. For clarity only the major disorder component of the disordered tert-butyl and propoxy groups are shown.

**Fig. 2.**
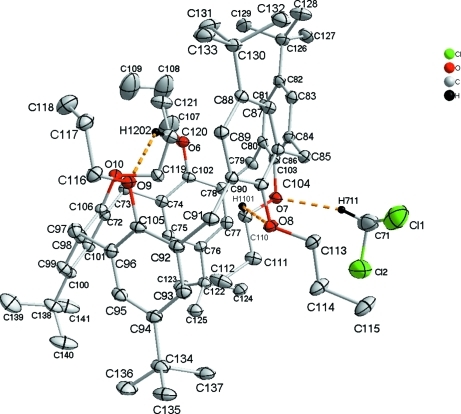
A view of second molecule of 1 with atom's numbering. Displacement ellipsoids are drawn at the 30% probability level. Hydrogen bonds are drawn as dashed lines. Hydrogen atoms not participating in hydrogen bonds are omitted. For clarity only the major disorder component of the disordered tert-butyl and propoxy groups are shown.

**Fig. 3.**
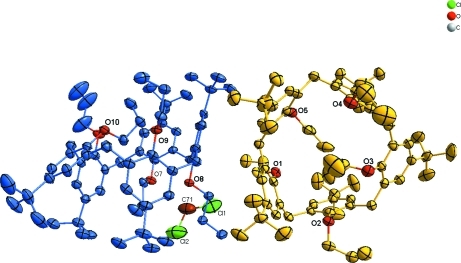
The asymmetric unit of 1.

## supplementary crystallographic information

### Comment

The studied compound was prepared by alkylation of *tert*-butylcalix[5]arene with propyl iodide in a presence of NaH and crystallized from mixture of dichloromethane and methanol. Molecules of dichloromethane are present in the crystal structure. The asymmetric unit contains two molecules of calix[5]arene which are turned to each other in direction of the *b* axis at an angle of 67/% (angle between planes formed by the bridging CH_2_ groups). This leads to the formation of infinite channels along the *a* axis by half of the molecules. The channels are separated by layers of turned molecules. The solvent molecule forms C—H···O hydrogen bonds. Hydrogen atom (H711) of the CH_2_ group in dichloromethane point toward O7, the distance between HC—H···O is 2.36 Å, and the chlorine atom has close contact to the terminal CH_3_ group of the propoxy chain (the HC—H···Cl distance is 2.92 Å). In each macrocycle two propoxy chains point above the cavity, while the others are turned out. The orientation toward the cavity is caused by intra-molecular hydrogen bonds between a methylene group of a propoxy chain and a neighbouring oxygen atom (C110-H1101···O8, C120-H1202···O9, C48—H482···O1, the distances are given in Table 1). Two disordered positions were modeled for three *tert*-butyl groups and the C atoms of two propoxy groups. The disorder components were found from difference electron density maps and their occupancy factors were fixed after several cycles of refinement as follows: *tert*-butyl groups with central carbon atoms: C67 - 0.59:0.40; C137 - 0.59:0.41; C138 - 0.85:0.15; propoxy group bound to oxygen atoms: O1 - 0.7372:0.2628 and O6 - 0.5027: 0.4973.

### Experimental

To the solution of 250 mg (0.3 mmol) of 1 in 15 ml of dry DMF cooled to 0 °C was added 123 mg (3 mmol) of 60% suspension of NaH in mineral oil and the mixture was stirred for 15 minutes. Then, 0.45 ml of propyl iodide was added and the reaction was stirred at room temperature. After two days, the mixture was poured into 30 ml of 1 *M* HCl and extracted 3 τimes 20 ml of dichloromethane. Combined extracts were washed with 20 ml of water and 20 ml of brine and dried over MgSO_4_. The drying agent was filtered off and the filtrate was evaporated to dryness. The crude product was dissolved in 10 ml of dichloromethane, 10 ml of MeOH was added and the mixture was slowly evaporated in an open flask. After two days, 235 mg (75%) of the white crystals of product 2 was obtained.

NMR spectra corresponded with data in the literature (Stewart *et al.*, 1995) - on a compound prepared using a different procedure, in acetonitrile with K_2_CO_3_, yield 83%. MS-ESI+: C_70_H_100_O_5_ required 1020.76; found 1043.71 ([*M*+Na]+; 100%), 1059.70 ([*M*+K]+; 10%)

### Refinement

Refinement of F^2^ against ALL reflections. The weighted *R*-factor *wR* and goodness of fit S are based on F^2^, conventional *R*-factors *R* are based on F, with F set to zero for negative F^2^. The threshold expression of F^2^ > 2sigma(F^2^) is used only for calculating *R*-factors(gt) *etc*. and is not relevant to the choice of reflections for refinement. *R*-factors based on F^2^ are statistically about twice as large as those based on F, and R– factors based on ALL data will be even larger. The position of disordered atoms were located from difference maps of electron density. Disordered fragments were then placed in appropriate positions, and all distances between neighbouring atoms and angles were restrained. Site occupancies were refined for the different parts with the same thermal parameters for the same atoms in the various fragments. At the end of refinement, site occupancies were fixed and hydrogen atoms were placed in calculated positions with the thermal parameters *U*_iso_(H) (in the range 1.2–1.5 times *U*_eq_ of the parent atom).

### Crystal data

**Table:**

C_70_H_100_O_5_·0.5CH_2_Cl_2_	*F*(000) = 4648
*M**_r_* = 1064.03	*D*_x_ = 1.095 Mg m^−^^3^
Monoclinic, *P*2_1_/*c*	Mo *K*α radiation, λ = 0.71073 Å
Hall symbol: -P 2ybc	Cell parameters from 390743 reflections
*a* = 12.3740 (2) Å	θ = 1.0–24.1°
*b* = 23.9130 (2) Å	µ = 0.11 mm^−^^1^
*c* = 43.6550 (5) Å	*T* = 150 K
β = 91.7460 (5)°	Plate, colourless
*V* = 12911.5 (3) Å^3^	0.45 × 0.23 × 0.12 mm
*Z* = 8	

### Data collection

**Table:**

Nonius KappaCCD diffractometer	*R*_int_ = 0.043
graphite	θ_max_ = 24.1°, θ_min_ = 1.3°
ω/2θ scans	*h* = −14→14
39073 measured reflections	*k* = −27→27
20282 independent reflections	*l* = −50→50
13395 reflections with *I* > 2σ(*I*)	

### Refinement

**Table:**

Refinement on *F*^2^	Primary atom site location: structure-invariant direct methods
Least-squares matrix: full	Hydrogen site location: inferred from neighbouring sites
*R*[*F*^2^ > 2σ(*F*^2^)] = 0.074	H-atom parameters constrained
*wR*(*F*^2^) = 0.151	Method, part 1, Chebychev polynomial, (Watkin, 1994, *P*rince, 1982) [*w*eight] = 1.0/[A_0_*T_0_(x) + A_1_*T_1_(x) ··· + A_n-1_]*T_n-1_(x)] where A_i_ are the Chebychev coefficients listed belo*w* and x = *F* /*F*max Method = Robust Weighting (*P*rince, 1982) W = [*w*eight] * [1-(delta*F*/6*sigma*F*)^2^]^2^ A_i_ are: 11.5 14.0 3.91
*S* = 1.02	(Δ/σ)_max_ = 0.001
20282 reflections	Δρ_max_ = 1.88 e Å^−^^3^
1531 parameters	Δρ_min_ = −1.75 e Å^−^^3^
220 restraints	

### Fractional atomic coordinates and isotropic or equivalent isotropic displacement parameters (Å^2^)

**Table:**

	*x*	*y*	*z*	*U*_iso_*/*U*_eq_	Occ. (<1)
Cl1	0.57648 (17)	0.44344 (8)	0.53618 (5)	0.1149	
Cl2	0.75149 (18)	0.42717 (11)	0.49435 (7)	0.1499	
O1	0.3168 (2)	0.36142 (11)	0.61877 (6)	0.0385	
O2	0.4804 (2)	0.27551 (11)	0.70453 (6)	0.0369	
O3	0.2037 (2)	0.23598 (12)	0.73657 (7)	0.0465	
O4	−0.0814 (2)	0.19447 (12)	0.69175 (7)	0.0446	
O5	0.0087 (2)	0.31590 (12)	0.62105 (6)	0.0396	
O7	0.4990 (2)	0.38058 (11)	0.44291 (6)	0.0350	
O8	0.44330 (19)	0.26652 (11)	0.50838 (6)	0.0313	
O9	0.21648 (19)	0.13254 (11)	0.46283 (6)	0.0317	
O10	0.2735 (2)	0.18753 (12)	0.38854 (6)	0.0394	
C1	0.0976 (3)	0.23706 (17)	0.59817 (9)	0.0331	
C2	0.1905 (3)	0.27406 (17)	0.58846 (9)	0.0354	
C3	0.2992 (3)	0.26365 (17)	0.60504 (9)	0.0327	
C4	0.3457 (3)	0.21050 (17)	0.60532 (9)	0.0344	
C5	0.4462 (3)	0.19915 (17)	0.61923 (9)	0.0343	
C6	0.4981 (3)	0.24336 (17)	0.63415 (9)	0.0336	
C7	0.4556 (3)	0.29704 (16)	0.63505 (9)	0.0324	
C8	0.5175 (3)	0.34373 (17)	0.65123 (9)	0.0369	
C9	0.4690 (3)	0.36604 (16)	0.68048 (9)	0.0321	
C10	0.4440 (3)	0.42264 (17)	0.68304 (9)	0.0366	
C11	0.3998 (3)	0.44602 (17)	0.70921 (9)	0.0366	
C12	0.3743 (3)	0.40857 (17)	0.73253 (9)	0.0367	
C13	0.3977 (3)	0.35146 (17)	0.73122 (9)	0.0331	
C14	0.3679 (3)	0.31298 (18)	0.75739 (9)	0.0368	
C15	0.2511 (3)	0.31707 (17)	0.76683 (9)	0.0349	
C16	0.2218 (3)	0.35663 (19)	0.78843 (9)	0.0410	
C17	0.1162 (3)	0.36135 (19)	0.79821 (9)	0.0421	
C18	0.0403 (3)	0.32465 (19)	0.78536 (9)	0.0422	
C19	0.0651 (3)	0.28482 (18)	0.76340 (10)	0.0379	
C20	−0.0241 (3)	0.24950 (19)	0.74867 (10)	0.0442	
C21	−0.0819 (3)	0.27921 (17)	0.72163 (10)	0.0368	
C22	−0.1100 (3)	0.33538 (17)	0.72356 (10)	0.0390	
C23	−0.1610 (3)	0.36469 (18)	0.69960 (9)	0.0363	
C24	−0.1785 (3)	0.33557 (17)	0.67210 (9)	0.0355	
C25	−0.1519 (3)	0.27880 (18)	0.66909 (10)	0.0376	
C26	−0.1724 (3)	0.2503 (2)	0.63880 (10)	0.0442	
C27	−0.0753 (3)	0.22581 (18)	0.62280 (9)	0.0366	
C28	−0.0759 (3)	0.17028 (18)	0.61437 (9)	0.0382	
C29	0.0059 (3)	0.14613 (18)	0.59739 (9)	0.0382	
C30	0.0907 (3)	0.18102 (17)	0.58940 (9)	0.0355	
C31	0.3570 (3)	0.30688 (16)	0.61932 (9)	0.0313	
C32	0.4498 (3)	0.33161 (16)	0.70573 (9)	0.0331	
C33	0.1723 (3)	0.28035 (17)	0.75489 (9)	0.0356	
C34	−0.1069 (3)	0.25127 (17)	0.69434 (10)	0.0373	
C35	0.0118 (3)	0.25910 (17)	0.61450 (9)	0.0345	
C39	0.5932 (3)	0.2661 (2)	0.71254 (11)	0.0482	
C40	0.6153 (4)	0.2544 (2)	0.74556 (11)	0.0555	
C41	0.5689 (5)	0.1992 (2)	0.75635 (14)	0.0754	
C42	0.2004 (4)	0.2471 (2)	0.70448 (10)	0.0486	
C43	0.2555 (6)	0.2000 (3)	0.68780 (14)	0.0846	
C44	0.2068 (7)	0.1459 (3)	0.6895 (2)	0.1284	
C45	−0.1665 (4)	0.1582 (2)	0.70146 (14)	0.0657	
C46	−0.1239 (5)	0.0991 (2)	0.70335 (16)	0.0791	
C47	−0.0469 (6)	0.0912 (3)	0.73034 (18)	0.1052	
C48	0.0753 (3)	0.33122 (17)	0.64732 (10)	0.0390	
C49	0.0738 (4)	0.39336 (19)	0.65105 (11)	0.0499	
C50	0.1414 (4)	0.4096 (3)	0.67903 (15)	0.0842	
C51	0.5002 (3)	0.14147 (18)	0.61738 (11)	0.0440	
C52	0.4236 (4)	0.09687 (19)	0.60426 (13)	0.0609	
C53	0.5965 (4)	0.1456 (2)	0.59599 (13)	0.0665	
C54	0.5408 (5)	0.1230 (2)	0.64941 (12)	0.0711	
C55	0.3829 (4)	0.50901 (18)	0.71227 (11)	0.0441	
C56	0.4919 (5)	0.5358 (2)	0.7190 (2)	0.1149	
C57	0.3044 (5)	0.5239 (2)	0.73684 (14)	0.0875	
C58	0.3378 (6)	0.5344 (2)	0.68244 (13)	0.0828	
C59	0.0816 (4)	0.4055 (2)	0.82133 (10)	0.0552	
C60	0.1754 (5)	0.4432 (3)	0.83163 (14)	0.0818	
C61	0.0371 (6)	0.3769 (3)	0.84946 (12)	0.0914	
C62	−0.0046 (4)	0.4437 (2)	0.80613 (12)	0.0727	
C63	−0.1956 (3)	0.42560 (18)	0.70422 (10)	0.0396	
C64	−0.0977 (4)	0.46152 (19)	0.71482 (12)	0.0556	
C65	−0.2798 (4)	0.4267 (2)	0.72898 (11)	0.0547	
C66	−0.2449 (4)	0.45182 (19)	0.67507 (10)	0.0477	
C71	0.6226 (5)	0.4543 (3)	0.49944 (17)	0.0921	
C72	0.4129 (3)	0.18398 (17)	0.35096 (9)	0.0337	
C73	0.3859 (3)	0.24204 (16)	0.33889 (9)	0.0360	
C74	0.4649 (3)	0.28711 (16)	0.35017 (9)	0.0331	
C75	0.5754 (3)	0.27735 (18)	0.35251 (9)	0.0368	
C76	0.6493 (3)	0.31754 (18)	0.36326 (9)	0.0366	
C77	0.6063 (3)	0.36744 (18)	0.37403 (9)	0.0386	
C78	0.4954 (3)	0.37921 (16)	0.37216 (9)	0.0325	
C79	0.4527 (3)	0.43374 (17)	0.38472 (10)	0.0401	
C80	0.3719 (3)	0.43022 (16)	0.41026 (9)	0.0329	
C81	0.2732 (3)	0.45755 (16)	0.40702 (10)	0.0377	
C82	0.1998 (3)	0.46128 (16)	0.43037 (9)	0.0350	
C83	0.2274 (3)	0.43444 (16)	0.45753 (10)	0.0353	
C84	0.3232 (3)	0.40413 (16)	0.46212 (9)	0.0305	
C85	0.3493 (3)	0.37538 (16)	0.49267 (9)	0.0335	
C86	0.2831 (3)	0.32317 (16)	0.49943 (8)	0.0287	
C87	0.1695 (3)	0.32567 (16)	0.49931 (9)	0.0318	
C88	0.1048 (3)	0.27963 (16)	0.50487 (9)	0.0308	
C89	0.1560 (3)	0.22889 (16)	0.51008 (8)	0.0308	
C90	0.2691 (3)	0.22374 (16)	0.51103 (8)	0.0285	
C91	0.3210 (3)	0.16770 (16)	0.51910 (9)	0.0314	
C92	0.3798 (3)	0.13757 (15)	0.49404 (9)	0.0292	
C93	0.4877 (3)	0.12282 (16)	0.49839 (9)	0.0328	
C94	0.5442 (3)	0.09268 (16)	0.47680 (10)	0.0342	
C95	0.4904 (3)	0.08084 (16)	0.44912 (10)	0.0350	
C96	0.3819 (3)	0.09491 (16)	0.44346 (9)	0.0335	
C97	0.3278 (3)	0.08105 (17)	0.41269 (9)	0.0375	
C98	0.3863 (3)	0.10652 (17)	0.38593 (9)	0.0352	
C99	0.4666 (3)	0.07706 (18)	0.37127 (9)	0.0394	
C100	0.5235 (3)	0.09963 (18)	0.34715 (9)	0.0386	
C101	0.4935 (3)	0.15263 (17)	0.33717 (9)	0.0364	
C102	0.4273 (3)	0.33965 (17)	0.35875 (9)	0.0338	
C103	0.3960 (3)	0.40346 (16)	0.43806 (9)	0.0314	
C104	0.3314 (3)	0.27171 (16)	0.50648 (8)	0.0300	
C105	0.3270 (3)	0.12129 (15)	0.46665 (9)	0.0295	
C106	0.3597 (3)	0.16010 (17)	0.37535 (9)	0.0340	
C110	0.5036 (3)	0.32053 (16)	0.43991 (9)	0.0331	
C111	0.6159 (3)	0.30165 (18)	0.44939 (10)	0.0429	
C112	0.6232 (4)	0.2383 (2)	0.44767 (13)	0.0632	
C113	0.4892 (3)	0.28464 (18)	0.53762 (9)	0.0356	
C114	0.6099 (3)	0.2761 (2)	0.53786 (10)	0.0507	
C115	0.6632 (3)	0.2964 (2)	0.56753 (10)	0.0601	
C116	0.1508 (3)	0.08509 (18)	0.46990 (10)	0.0382	
C117	0.0337 (3)	0.1005 (2)	0.46478 (10)	0.0458	
C118	0.0045 (4)	0.1146 (2)	0.43170 (12)	0.0660	
C119	0.3045 (3)	0.23067 (17)	0.41036 (9)	0.0374	
C120	0.2040 (3)	0.25435 (18)	0.42402 (10)	0.0411	
C121	0.1302 (4)	0.2835 (2)	0.40130 (11)	0.0589	
C122	0.7708 (3)	0.30570 (19)	0.36255 (11)	0.0442	
C123	0.8015 (4)	0.2962 (2)	0.32900 (12)	0.0616	
C124	0.8383 (4)	0.3538 (2)	0.37563 (13)	0.0609	
C125	0.7994 (4)	0.2530 (2)	0.38076 (12)	0.0546	
C126	0.0947 (3)	0.49451 (18)	0.42547 (11)	0.0438	
C127	0.1222 (4)	0.55546 (18)	0.41810 (11)	0.0509	
C128	0.0246 (4)	0.4942 (2)	0.45379 (12)	0.0617	
C129	0.0298 (4)	0.4691 (2)	0.39812 (13)	0.0670	
C130	−0.0185 (3)	0.28697 (17)	0.50565 (10)	0.0370	
C131	−0.0611 (3)	0.3027 (2)	0.47376 (11)	0.0600	
C132	−0.0454 (3)	0.33372 (19)	0.52823 (11)	0.0509	
C133	−0.0763 (3)	0.23437 (19)	0.51616 (12)	0.0532	
C134	0.6598 (3)	0.07164 (18)	0.48388 (12)	0.0484	
C135	0.6575 (6)	0.0370 (3)	0.51548 (18)	0.0476	0.5901
C136	0.7090 (7)	0.0358 (4)	0.4610 (2)	0.0596	0.5901
C137	0.7350 (6)	0.1218 (3)	0.4924 (2)	0.0517	0.5901
C335	0.6565 (11)	0.0080 (5)	0.4800 (4)	0.0955	0.4099
C336	0.7242 (10)	0.0847 (8)	0.4528 (3)	0.0975	0.4099
C337	0.7146 (12)	0.0991 (9)	0.5083 (4)	0.1125	0.4099
O6	0.3176 (2)	0.35209 (12)	0.35529 (7)	0.0415	
C107	0.2872 (16)	0.3702 (12)	0.3248 (4)	0.1249	0.5027
C108	0.1665 (15)	0.3862 (7)	0.3215 (5)	0.1379	0.5027
C109	0.1217 (16)	0.3326 (7)	0.3150 (4)	0.1466	0.5027
C207	0.2826 (8)	0.3635 (7)	0.3245 (3)	0.0407	0.4973
C208	0.1594 (8)	0.3581 (5)	0.3250 (3)	0.0586	0.4973
C209	0.1099 (9)	0.3696 (4)	0.2938 (2)	0.0636	0.4973
C138	0.6146 (8)	0.0672 (3)	0.33234 (19)	0.0577	0.8535
C139	0.5640 (7)	0.0173 (3)	0.3152 (2)	0.1007	0.8535
C140	0.6942 (6)	0.0454 (4)	0.35689 (16)	0.0883	0.8535
C141	0.6763 (7)	0.1023 (4)	0.30980 (17)	0.0876	0.8535
C338	0.609 (3)	0.0655 (14)	0.3296 (8)	0.0460	0.1465
C339	0.559 (2)	0.0436 (12)	0.2993 (5)	0.0399	0.1465
C340	0.648 (3)	0.0148 (12)	0.3481 (6)	0.0422	0.1465
C341	0.703 (3)	0.1045 (14)	0.3230 (7)	0.0439	0.1465
C67	0.0038 (8)	0.0843 (7)	0.5877 (3)	0.0350	0.5917
C68	−0.0996 (8)	0.0536 (5)	0.5973 (2)	0.0529	0.5917
C69	0.0100 (8)	0.0801 (4)	0.5524 (2)	0.0561	0.5917
C70	0.1009 (8)	0.0532 (5)	0.6022 (3)	0.0657	0.5917
C267	0.0053 (17)	0.0842 (12)	0.5897 (5)	0.0650	0.4083
C268	−0.1104 (14)	0.0634 (8)	0.5840 (4)	0.0805	0.4083
C269	0.0613 (15)	0.0728 (7)	0.5598 (4)	0.0850	0.4083
C270	0.0613 (16)	0.0525 (7)	0.6163 (4)	0.0847	0.4083
C36	0.3429 (9)	0.3941 (3)	0.59245 (19)	0.0423	0.7372
C37	0.2926 (7)	0.4505 (3)	0.59579 (18)	0.0539	0.7372
C38	0.3076 (6)	0.4872 (3)	0.56818 (15)	0.0600	0.7372
C236	0.326 (3)	0.3806 (10)	0.5872 (5)	0.0432	0.2628
C237	0.2568 (16)	0.4308 (7)	0.5791 (4)	0.0514	0.2628
C238	0.281 (2)	0.4777 (8)	0.6015 (5)	0.0620	0.2628
H21	0.1701	0.3148	0.5916	0.0471*	
H22	0.2013	0.2685	0.5659	0.0474*	
H41	0.3062	0.1796	0.5959	0.0466*	
H61	0.5684	0.2370	0.6444	0.0453*	
H81	0.5922	0.3297	0.6567	0.0501*	
H82	0.5233	0.3760	0.6366	0.0500*	
H101	0.4579	0.4474	0.6656	0.0494*	
H121	0.3373	0.4227	0.7508	0.0494*	
H141	0.4159	0.3227	0.7756	0.0502*	
H142	0.3839	0.2733	0.7511	0.0504*	
H161	0.2777	0.3824	0.7968	0.0556*	
H181	−0.0346	0.3264	0.7922	0.0551*	
H202	−0.0793	0.2406	0.7643	0.0595*	
H201	0.0074	0.2130	0.7414	0.0594*	
H221	−0.0943	0.3554	0.7430	0.0529*	
H241	−0.2099	0.3556	0.6540	0.0495*	
H261	−0.2229	0.2180	0.6423	0.0594*	
H262	−0.2087	0.2777	0.6249	0.0592*	
H281	−0.1373	0.1466	0.6205	0.0522*	
H301	0.1482	0.1654	0.5774	0.0465*	
H392	0.6374	0.3003	0.7063	0.0611*	
H391	0.6167	0.2328	0.7004	0.0615*	
H401	0.5827	0.2860	0.7577	0.0734*	
H402	0.6967	0.2540	0.7498	0.0733*	
H413	0.5874	0.1926	0.7782	0.1181*	
H412	0.4901	0.1984	0.7530	0.1179*	
H411	0.6004	0.1682	0.7440	0.1183*	
H422	0.2395	0.2851	0.7013	0.0648*	
H421	0.1233	0.2516	0.6966	0.0644*	
H431	0.3335	0.1954	0.6964	0.1075*	
H432	0.2590	0.2093	0.6647	0.1077*	
H441	0.2456	0.1187	0.6768	0.1933*	
H443	0.2154	0.1339	0.7120	0.1936*	
H442	0.1308	0.1489	0.6836	0.1932*	
H451	−0.1949	0.1702	0.7221	0.0873*	
H452	−0.2289	0.1603	0.6859	0.0874*	
H462	−0.1853	0.0716	0.7050	0.1030*	
H461	−0.0847	0.0905	0.6840	0.1034*	
H471	−0.0260	0.0517	0.7324	0.1573*	
H473	−0.0833	0.1035	0.7496	0.1573*	
H472	0.0189	0.1144	0.7279	0.1570*	
H481	0.0461	0.3129	0.6664	0.0532*	
H482	0.1509	0.3182	0.6452	0.0526*	
H492	−0.0028	0.4068	0.6531	0.0669*	
H491	0.1045	0.4109	0.6323	0.0677*	
H501	0.1380	0.4503	0.6823	0.1272*	
H502	0.1122	0.3909	0.6976	0.1272*	
H503	0.2169	0.3976	0.6765	0.1273*	
H522	0.4595	0.0603	0.6040	0.0990*	
H521	0.3598	0.0945	0.6172	0.0991*	
H523	0.3998	0.1067	0.5829	0.0991*	
H532	0.6319	0.1093	0.5942	0.1030*	
H531	0.6503	0.1725	0.6043	0.1032*	
H533	0.5716	0.1584	0.5756	0.1034*	
H543	0.5774	0.0864	0.6482	0.1111*	
H542	0.5940	0.1510	0.6580	0.1112*	
H541	0.4795	0.1202	0.6631	0.1110*	
H563	0.4835	0.5766	0.7206	0.1702*	
H562	0.5196	0.5211	0.7389	0.1702*	
H561	0.5416	0.5262	0.7030	0.1702*	
H571	0.2898	0.5640	0.7370	0.1418*	
H572	0.3354	0.5134	0.7571	0.1422*	
H573	0.2357	0.5037	0.7340	0.1421*	
H582	0.3274	0.5745	0.6851	0.1289*	
H581	0.2677	0.5158	0.6766	0.1296*	
H583	0.3889	0.5277	0.6658	0.1293*	
H602	0.1477	0.4714	0.8464	0.1233*	
H601	0.2344	0.4217	0.8419	0.1230*	
H603	0.2055	0.4639	0.8138	0.1233*	
H613	0.0185	0.4051	0.8646	0.1412*	
H612	0.0888	0.3504	0.8580	0.1410*	
H611	−0.0302	0.3561	0.8430	0.1411*	
H623	−0.0245	0.4733	0.8202	0.1209*	
H622	−0.0693	0.4215	0.8000	0.1210*	
H621	0.0231	0.4616	0.7874	0.1215*	
H641	−0.1237	0.4995	0.7203	0.0921*	
H642	−0.0618	0.4447	0.7330	0.0920*	
H643	−0.0455	0.4649	0.6978	0.0924*	
H652	−0.3016	0.4662	0.7327	0.0903*	
H651	−0.2488	0.4111	0.7485	0.0904*	
H653	−0.3437	0.4053	0.7225	0.0900*	
H662	−0.2643	0.4913	0.6790	0.0779*	
H661	−0.3108	0.4313	0.6683	0.0779*	
H663	−0.1920	0.4508	0.6587	0.0783*	
H712	0.6226	0.4954	0.4944	0.1125*	
H711	0.5706	0.4357	0.4836	0.1122*	
H731	0.3118	0.2524	0.3452	0.0471*	
H732	0.3849	0.2406	0.3159	0.0472*	
H751	0.6030	0.2408	0.3464	0.0485*	
H771	0.6540	0.3954	0.3827	0.0513*	
H791	0.5152	0.4568	0.3920	0.0545*	
H792	0.4155	0.4541	0.3669	0.0544*	
H811	0.2562	0.4752	0.3875	0.0513*	
H831	0.1790	0.4374	0.4744	0.0475*	
H851	0.3371	0.4030	0.5096	0.0452*	
H852	0.4294	0.3653	0.4934	0.0450*	
H871	0.1345	0.3615	0.4952	0.0425*	
H891	0.1127	0.1958	0.5130	0.0424*	
H911	0.3753	0.1735	0.5367	0.0441*	
H912	0.2643	0.1423	0.5266	0.0440*	
H931	0.5247	0.1349	0.5174	0.0446*	
H951	0.5289	0.0616	0.4331	0.0476*	
H972	0.2515	0.0952	0.4122	0.0492*	
H971	0.3257	0.0394	0.4099	0.0493*	
H991	0.4843	0.0389	0.3787	0.0513*	
H1011	0.5313	0.1694	0.3198	0.0504*	
H1101	0.4484	0.3033	0.4532	0.0450*	
H1102	0.4855	0.3097	0.4177	0.0449*	
H1112	0.6311	0.3152	0.4710	0.0582*	
H1111	0.6696	0.3195	0.4356	0.0582*	
H1123	0.6962	0.2252	0.4541	0.1011*	
H1122	0.5718	0.2209	0.4616	0.1010*	
H1121	0.6063	0.2242	0.4269	0.1009*	
H1131	0.4553	0.2636	0.5548	0.0497*	
H1132	0.4742	0.3260	0.5406	0.0493*	
H1142	0.6257	0.2347	0.5353	0.0667*	
H1141	0.6397	0.2965	0.5199	0.0662*	
H1153	0.7391	0.2886	0.5680	0.0958*	
H1152	0.6300	0.2784	0.5854	0.0962*	
H1151	0.6523	0.3377	0.5691	0.0963*	
H1161	0.1661	0.0727	0.4925	0.0508*	
H1162	0.1695	0.0535	0.4559	0.0503*	
H1171	0.0177	0.1339	0.4777	0.0607*	
H1172	−0.0147	0.0690	0.4718	0.0605*	
H1182	−0.0724	0.1248	0.4294	0.1040*	
H1181	0.0488	0.1459	0.4249	0.1040*	
H1183	0.0191	0.0821	0.4188	0.1044*	
H1192	0.3539	0.2144	0.4272	0.0504*	
H1191	0.3451	0.2609	0.3994	0.0500*	
H1201	0.2261	0.2824	0.4408	0.0557*	
H1202	0.1647	0.2232	0.4342	0.0554*	
H1212	0.0670	0.2991	0.4113	0.0952*	
H1211	0.1695	0.3145	0.3916	0.0950*	
H1213	0.1071	0.2572	0.3853	0.0952*	
H1232	0.8788	0.2879	0.3280	0.0989*	
H1231	0.7852	0.3303	0.3169	0.0992*	
H1233	0.7585	0.2649	0.3198	0.0994*	
H1241	0.9145	0.3453	0.3737	0.1020*	
H1243	0.8213	0.3600	0.3970	0.1019*	
H1242	0.8214	0.3886	0.3637	0.1020*	
H1251	0.8756	0.2442	0.3787	0.0908*	
H1253	0.7861	0.2599	0.4025	0.0910*	
H1252	0.7551	0.2209	0.3734	0.0911*	
H1271	0.0558	0.5783	0.4160	0.0832*	
H1272	0.1684	0.5712	0.4350	0.0832*	
H1273	0.1629	0.5580	0.3990	0.0831*	
H1282	−0.0423	0.5155	0.4497	0.0959*	
H1281	0.0651	0.5107	0.4716	0.0961*	
H1283	0.0039	0.4555	0.4588	0.0962*	
H1292	−0.0375	0.4898	0.3948	0.1129*	
H1291	0.0121	0.4303	0.4028	0.1131*	
H1293	0.0719	0.4703	0.3793	0.1133*	
H1311	−0.1395	0.3084	0.4733	0.0978*	
H1313	−0.0272	0.3380	0.4671	0.0982*	
H1312	−0.0439	0.2727	0.4592	0.0981*	
H1321	−0.1242	0.3375	0.5305	0.0829*	
H1323	−0.0125	0.3258	0.5489	0.0832*	
H1322	−0.0180	0.3694	0.5208	0.0829*	
H1331	−0.1536	0.2405	0.5171	0.0869*	
H1332	−0.0487	0.2221	0.5367	0.0872*	
H1333	−0.0635	0.2035	0.5015	0.0870*	
H1352	0.7276	0.0226	0.5209	0.0789*	0.5901
H1351	0.6355	0.0604	0.5314	0.0789*	0.5901
H1353	0.6085	0.0062	0.5133	0.0791*	0.5901
H1362	0.7792	0.0216	0.4684	0.0938*	0.5901
H1361	0.6612	0.0051	0.4555	0.0939*	0.5901
H1363	0.7221	0.0582	0.4422	0.0941*	0.5901
H1371	0.8032	0.1085	0.5017	0.0882*	0.5901
H1373	0.6995	0.1479	0.5061	0.0883*	0.5901
H1372	0.7518	0.1414	0.4734	0.0878*	0.5901
H3351	0.7291	−0.0060	0.4769	0.1489*	0.4099
H3353	0.6300	−0.0081	0.4988	0.1491*	0.4099
H3352	0.6105	−0.0019	0.4632	0.1489*	0.4099
H3361	0.7941	0.0679	0.4532	0.1479*	0.4099
H3362	0.7297	0.1238	0.4493	0.1479*	0.4099
H3363	0.6822	0.0684	0.4349	0.1480*	0.4099
H3372	0.7934	0.1066	0.5021	0.1601*	0.4099
H3371	0.7097	0.0870	0.5269	0.1601*	0.4099
H3373	0.6859	0.1419	0.5072	0.1602*	0.4099
H1072	0.3361	0.4039	0.3196	0.1390*	0.5027
H1071	0.3079	0.3411	0.3092	0.1392*	0.5027
H1081	0.1431	0.4039	0.3405	0.1502*	0.5027
H1082	0.1555	0.4113	0.3041	0.1502*	0.5027
H1093	0.0468	0.3375	0.3115	0.1931*	0.5027
H1092	0.1352	0.3082	0.3333	0.1932*	0.5027
H1091	0.1560	0.3145	0.2977	0.1932*	0.5027
H2072	0.3037	0.4000	0.3179	0.0561*	0.4973
H2071	0.3084	0.3344	0.3099	0.0562*	0.4973
H2081	0.1253	0.3857	0.3396	0.0739*	0.4973
H2082	0.1386	0.3209	0.3324	0.0744*	0.4973
H2093	0.0320	0.3727	0.2951	0.1000*	0.4973
H2092	0.1385	0.4037	0.2859	0.0999*	0.4973
H2091	0.1280	0.3399	0.2803	0.1000*	0.4973
H1393	0.6223	−0.0048	0.3063	0.1501*	0.8535
H1392	0.5253	−0.0063	0.3295	0.1498*	0.8535
H1391	0.5146	0.0308	0.2987	0.1501*	0.8535
H1401	0.7541	0.0258	0.3474	0.1381*	0.8535
H1402	0.7240	0.0778	0.3687	0.1380*	0.8535
H1403	0.6583	0.0199	0.3706	0.1380*	0.8535
H1412	0.7362	0.0817	0.3017	0.1350*	0.8535
H1411	0.7055	0.1361	0.3205	0.1354*	0.8535
H1413	0.6280	0.1143	0.2926	0.1350*	0.8535
H3392	0.6270	0.0260	0.2862	0.0670*	0.1465
H3391	0.4987	0.0108	0.3058	0.0670*	0.1465
H3393	0.5193	0.0799	0.2872	0.0670*	0.1465
H3403	0.7193	0.0039	0.3397	0.0620*	0.1465
H3402	0.6670	0.0291	0.3689	0.0620*	0.1465
H3401	0.6020	−0.0131	0.3481	0.0619*	0.1465
H3413	0.7492	0.0883	0.3077	0.0640*	0.1465
H3412	0.7424	0.1142	0.3406	0.0640*	0.1465
H3411	0.6738	0.1400	0.3132	0.0641*	0.1465
H681	−0.1012	0.0160	0.5886	0.0809*	0.5917
H682	−0.1030	0.0514	0.6190	0.0809*	0.5917
H683	−0.1646	0.0745	0.5892	0.0815*	0.5917
H692	0.0125	0.0413	0.5465	0.0860*	0.5917
H691	0.0757	0.0995	0.5452	0.0864*	0.5917
H693	−0.0527	0.0978	0.5431	0.0858*	0.5917
H702	0.0910	0.0127	0.5994	0.0950*	0.5917
H701	0.1062	0.0607	0.6244	0.0955*	0.5917
H703	0.1651	0.0644	0.5931	0.0948*	0.5917
H2683	−0.1154	0.0277	0.5763	0.1291*	0.4083
H2682	−0.1520	0.0667	0.6037	0.1292*	0.4083
H2681	−0.1488	0.0907	0.5695	0.1291*	0.4083
H2693	0.0404	0.0386	0.5512	0.1300*	0.4083
H2692	0.1388	0.0735	0.5647	0.1299*	0.4083
H2691	0.0447	0.1043	0.5455	0.1302*	0.4083
H2701	0.0589	0.0134	0.6139	0.1320*	0.4083
H2702	0.0203	0.0616	0.6357	0.1320*	0.4083
H2703	0.1342	0.0664	0.6196	0.1321*	0.4083
H362	0.4224	0.3981	0.5909	0.0560*	0.7372
H361	0.3157	0.3759	0.5730	0.0564*	0.7372
H372	0.3236	0.4692	0.6148	0.0743*	0.7372
H371	0.2138	0.4460	0.5991	0.0742*	0.7372
H381	0.2806	0.5253	0.5714	0.0941*	0.7372
H382	0.3834	0.4894	0.5634	0.0939*	0.7372
H383	0.2679	0.4718	0.5502	0.0941*	0.7372
H2362	0.4007	0.3860	0.5822	0.0560*	0.2628
H2361	0.2975	0.3488	0.5734	0.0561*	0.2628
H2372	0.1831	0.4217	0.5817	0.0740*	0.2628
H2371	0.2724	0.4428	0.5592	0.0740*	0.2628
H2381	0.2407	0.5111	0.5956	0.0960*	0.2628
H2382	0.2671	0.4686	0.6222	0.0959*	0.2628
H2383	0.3595	0.4885	0.6006	0.0961*	0.2628

### Atomic displacement parameters (Å^2^)

**Table:**

	*U*^11^	*U*^22^	*U*^33^	*U*^12^	*U*^13^	*U*^23^
Cl1	0.1092 (15)	0.0894 (13)	0.1436 (19)	−0.0272 (11)	−0.0370 (13)	0.0077 (13)
Cl2	0.0935 (15)	0.1327 (19)	0.221 (3)	0.0327 (14)	−0.0447 (16)	−0.0620 (19)
O1	0.0387 (16)	0.0324 (16)	0.0447 (17)	0.0002 (13)	0.0078 (13)	0.0051 (13)
O2	0.0309 (15)	0.0334 (16)	0.0466 (17)	0.0001 (12)	0.0045 (13)	0.0025 (13)
O3	0.0501 (18)	0.0413 (18)	0.0481 (18)	0.0043 (15)	0.0009 (15)	−0.0003 (15)
O4	0.0384 (16)	0.0338 (16)	0.062 (2)	0.0005 (13)	0.0082 (14)	−0.0016 (14)
O5	0.0390 (16)	0.0392 (17)	0.0405 (16)	0.0045 (13)	−0.0007 (13)	−0.0036 (13)
O7	0.0274 (14)	0.0287 (15)	0.0490 (17)	0.0011 (12)	0.0044 (12)	−0.0006 (13)
O8	0.0205 (13)	0.0413 (16)	0.0322 (14)	0.0012 (11)	0.0003 (11)	−0.0006 (12)
O9	0.0224 (13)	0.0329 (15)	0.0396 (16)	0.0037 (11)	−0.0011 (11)	−0.0002 (12)
O10	0.0292 (15)	0.0412 (17)	0.0479 (17)	0.0023 (13)	0.0032 (13)	−0.0113 (14)
C1	0.030 (2)	0.041 (2)	0.028 (2)	0.0029 (18)	−0.0029 (16)	0.0001 (18)
C2	0.036 (2)	0.037 (2)	0.033 (2)	0.0038 (18)	−0.0005 (18)	−0.0018 (18)
C3	0.030 (2)	0.036 (2)	0.032 (2)	0.0023 (18)	0.0068 (17)	0.0053 (18)
C4	0.033 (2)	0.035 (2)	0.035 (2)	−0.0040 (18)	0.0012 (17)	−0.0018 (18)
C5	0.033 (2)	0.032 (2)	0.038 (2)	−0.0001 (18)	0.0041 (18)	0.0030 (18)
C6	0.025 (2)	0.043 (2)	0.033 (2)	−0.0012 (18)	0.0042 (17)	0.0017 (18)
C7	0.031 (2)	0.035 (2)	0.031 (2)	−0.0034 (18)	0.0099 (17)	0.0005 (17)
C8	0.032 (2)	0.037 (2)	0.043 (2)	−0.0042 (18)	0.0081 (18)	0.0001 (19)
C9	0.028 (2)	0.033 (2)	0.035 (2)	−0.0044 (17)	0.0032 (17)	0.0000 (18)
C10	0.034 (2)	0.038 (2)	0.039 (2)	−0.0049 (18)	0.0049 (18)	0.0045 (19)
C11	0.035 (2)	0.036 (2)	0.039 (2)	−0.0004 (18)	0.0013 (18)	−0.0018 (19)
C12	0.034 (2)	0.040 (2)	0.037 (2)	0.0018 (19)	0.0048 (18)	0.0012 (19)
C13	0.026 (2)	0.035 (2)	0.038 (2)	−0.0036 (17)	0.0012 (17)	0.0027 (18)
C14	0.033 (2)	0.046 (3)	0.032 (2)	−0.0020 (19)	0.0019 (17)	0.0035 (19)
C15	0.035 (2)	0.039 (2)	0.031 (2)	0.0003 (19)	0.0007 (17)	0.0081 (18)
C16	0.040 (2)	0.044 (3)	0.039 (2)	0.003 (2)	0.0010 (19)	0.003 (2)
C17	0.041 (2)	0.054 (3)	0.031 (2)	0.011 (2)	0.0028 (19)	0.004 (2)
C18	0.036 (2)	0.055 (3)	0.036 (2)	0.006 (2)	0.0056 (19)	0.007 (2)
C19	0.033 (2)	0.038 (2)	0.042 (2)	−0.0002 (19)	0.0030 (19)	0.009 (2)
C20	0.042 (3)	0.044 (3)	0.047 (3)	−0.001 (2)	−0.001 (2)	0.012 (2)
C21	0.027 (2)	0.037 (2)	0.046 (3)	−0.0027 (18)	0.0021 (18)	0.001 (2)
C22	0.036 (2)	0.036 (2)	0.044 (3)	−0.0050 (19)	0.0028 (19)	0.003 (2)
C23	0.032 (2)	0.041 (2)	0.036 (2)	−0.0050 (18)	0.0014 (18)	0.0014 (19)
C24	0.024 (2)	0.043 (2)	0.039 (2)	−0.0006 (18)	0.0011 (17)	0.0037 (19)
C25	0.025 (2)	0.044 (3)	0.044 (2)	−0.0025 (18)	0.0043 (18)	−0.003 (2)
C26	0.032 (2)	0.052 (3)	0.049 (3)	0.003 (2)	0.003 (2)	−0.007 (2)
C27	0.026 (2)	0.046 (3)	0.038 (2)	0.0047 (19)	−0.0004 (17)	−0.002 (2)
C28	0.029 (2)	0.044 (3)	0.042 (2)	0.0006 (19)	0.0000 (18)	−0.002 (2)
C29	0.034 (2)	0.043 (2)	0.037 (2)	−0.0002 (19)	−0.0024 (18)	−0.002 (2)
C30	0.027 (2)	0.042 (2)	0.037 (2)	0.0035 (18)	0.0019 (17)	−0.0028 (19)
C31	0.032 (2)	0.031 (2)	0.031 (2)	0.0010 (17)	0.0087 (17)	0.0021 (17)
C32	0.027 (2)	0.032 (2)	0.040 (2)	−0.0016 (17)	0.0012 (17)	−0.0008 (18)
C33	0.039 (2)	0.036 (2)	0.032 (2)	0.0056 (19)	0.0045 (18)	0.0074 (18)
C34	0.024 (2)	0.032 (2)	0.056 (3)	−0.0026 (17)	0.0074 (19)	0.002 (2)
C35	0.033 (2)	0.036 (2)	0.033 (2)	0.0063 (18)	−0.0060 (17)	−0.0020 (18)
C39	0.033 (2)	0.047 (3)	0.064 (3)	0.005 (2)	0.002 (2)	0.003 (2)
C40	0.042 (3)	0.065 (3)	0.060 (3)	0.005 (2)	0.002 (2)	0.004 (3)
C41	0.072 (4)	0.070 (4)	0.085 (4)	0.015 (3)	0.009 (3)	0.032 (3)
C42	0.049 (3)	0.052 (3)	0.045 (3)	−0.001 (2)	0.004 (2)	0.000 (2)
C43	0.107 (5)	0.076 (4)	0.071 (4)	−0.003 (4)	0.022 (4)	−0.024 (3)
C44	0.130 (7)	0.089 (6)	0.169 (8)	−0.014 (5)	0.048 (6)	−0.058 (6)
C45	0.050 (3)	0.042 (3)	0.106 (5)	−0.009 (2)	0.006 (3)	0.000 (3)
C46	0.089 (5)	0.045 (3)	0.104 (5)	−0.006 (3)	0.015 (4)	0.002 (3)
C47	0.104 (6)	0.053 (4)	0.158 (7)	0.007 (4)	0.004 (5)	0.024 (4)
C48	0.031 (2)	0.042 (3)	0.044 (2)	−0.0002 (19)	0.0057 (19)	−0.003 (2)
C49	0.037 (2)	0.045 (3)	0.069 (3)	−0.006 (2)	0.007 (2)	−0.008 (2)
C50	0.044 (3)	0.082 (4)	0.127 (6)	−0.001 (3)	−0.005 (3)	−0.049 (4)
C51	0.040 (2)	0.038 (2)	0.053 (3)	0.007 (2)	−0.007 (2)	−0.005 (2)
C52	0.056 (3)	0.033 (3)	0.093 (4)	0.003 (2)	−0.013 (3)	−0.005 (3)
C53	0.047 (3)	0.050 (3)	0.104 (5)	0.010 (2)	0.017 (3)	−0.018 (3)
C54	0.087 (4)	0.050 (3)	0.075 (4)	0.018 (3)	−0.022 (3)	0.004 (3)
C55	0.045 (3)	0.034 (2)	0.054 (3)	0.000 (2)	0.009 (2)	−0.002 (2)
C56	0.074 (4)	0.043 (4)	0.227 (9)	−0.003 (3)	−0.004 (5)	−0.035 (5)
C57	0.126 (6)	0.047 (3)	0.093 (5)	0.027 (3)	0.055 (4)	0.005 (3)
C58	0.137 (6)	0.039 (3)	0.073 (4)	0.016 (3)	0.020 (4)	0.014 (3)
C59	0.055 (3)	0.075 (4)	0.035 (3)	0.022 (3)	−0.005 (2)	−0.009 (2)
C60	0.076 (4)	0.088 (4)	0.080 (4)	0.034 (3)	−0.026 (3)	−0.045 (4)
C61	0.123 (6)	0.112 (5)	0.040 (3)	0.041 (5)	0.023 (3)	−0.002 (3)
C62	0.070 (4)	0.084 (4)	0.063 (4)	0.039 (3)	−0.004 (3)	−0.013 (3)
C63	0.042 (2)	0.038 (2)	0.038 (2)	0.002 (2)	0.0002 (19)	0.0030 (19)
C64	0.059 (3)	0.037 (3)	0.070 (3)	−0.001 (2)	−0.009 (3)	−0.001 (2)
C65	0.058 (3)	0.052 (3)	0.055 (3)	0.012 (2)	0.012 (2)	0.000 (2)
C66	0.055 (3)	0.042 (3)	0.046 (3)	0.006 (2)	0.002 (2)	0.006 (2)
C71	0.065 (4)	0.067 (4)	0.144 (7)	−0.001 (3)	−0.014 (4)	−0.006 (4)
C72	0.032 (2)	0.036 (2)	0.034 (2)	0.0034 (18)	−0.0007 (17)	−0.0058 (18)
C73	0.034 (2)	0.035 (2)	0.038 (2)	0.0033 (18)	−0.0012 (18)	−0.0015 (19)
C74	0.035 (2)	0.033 (2)	0.031 (2)	0.0009 (18)	0.0052 (17)	0.0052 (18)
C75	0.034 (2)	0.038 (2)	0.039 (2)	0.0061 (19)	0.0022 (18)	−0.0002 (19)
C76	0.030 (2)	0.042 (2)	0.038 (2)	−0.0001 (19)	0.0016 (18)	0.0031 (19)
C77	0.039 (2)	0.038 (2)	0.039 (2)	−0.0038 (19)	0.0042 (19)	0.0030 (19)
C78	0.038 (2)	0.032 (2)	0.028 (2)	0.0006 (18)	0.0038 (17)	0.0029 (17)
C79	0.046 (3)	0.030 (2)	0.045 (3)	0.0020 (19)	0.011 (2)	0.0043 (19)
C80	0.037 (2)	0.027 (2)	0.034 (2)	−0.0016 (18)	0.0049 (18)	−0.0012 (17)
C81	0.041 (2)	0.029 (2)	0.043 (2)	0.0024 (19)	0.0002 (19)	0.0014 (19)
C82	0.034 (2)	0.028 (2)	0.043 (2)	0.0016 (17)	0.0018 (19)	−0.0005 (18)
C83	0.032 (2)	0.029 (2)	0.045 (2)	−0.0021 (18)	0.0045 (18)	−0.0027 (19)
C84	0.029 (2)	0.028 (2)	0.034 (2)	−0.0016 (17)	−0.0022 (17)	0.0001 (17)
C85	0.029 (2)	0.032 (2)	0.040 (2)	−0.0011 (17)	0.0003 (17)	−0.0047 (18)
C86	0.028 (2)	0.030 (2)	0.028 (2)	−0.0014 (16)	0.0008 (16)	−0.0031 (16)
C87	0.028 (2)	0.032 (2)	0.036 (2)	0.0042 (17)	0.0019 (17)	−0.0036 (18)
C88	0.025 (2)	0.036 (2)	0.031 (2)	0.0031 (17)	0.0042 (16)	−0.0058 (17)
C89	0.026 (2)	0.034 (2)	0.033 (2)	−0.0015 (17)	0.0030 (16)	−0.0034 (18)
C90	0.0247 (19)	0.033 (2)	0.028 (2)	0.0019 (16)	0.0007 (15)	−0.0045 (17)
C91	0.028 (2)	0.033 (2)	0.034 (2)	0.0016 (17)	0.0038 (17)	0.0025 (17)
C92	0.026 (2)	0.027 (2)	0.035 (2)	0.0015 (16)	0.0042 (16)	0.0043 (17)
C93	0.031 (2)	0.032 (2)	0.034 (2)	0.0012 (17)	0.0001 (17)	0.0037 (18)
C94	0.023 (2)	0.031 (2)	0.049 (3)	−0.0007 (17)	0.0051 (18)	0.0054 (19)
C95	0.030 (2)	0.030 (2)	0.046 (3)	0.0012 (17)	0.0121 (19)	0.0007 (19)
C96	0.031 (2)	0.030 (2)	0.039 (2)	−0.0020 (17)	0.0026 (18)	0.0029 (18)
C97	0.036 (2)	0.037 (2)	0.040 (2)	−0.0034 (19)	0.0060 (19)	−0.0026 (19)
C98	0.037 (2)	0.034 (2)	0.035 (2)	0.0007 (18)	0.0035 (18)	−0.0034 (18)
C99	0.045 (3)	0.036 (2)	0.038 (2)	0.007 (2)	0.010 (2)	−0.0018 (19)
C100	0.038 (2)	0.041 (2)	0.037 (2)	0.0096 (19)	0.0062 (19)	−0.0026 (19)
C101	0.040 (2)	0.039 (2)	0.031 (2)	0.0028 (19)	0.0043 (18)	−0.0022 (18)
C102	0.030 (2)	0.035 (2)	0.037 (2)	0.0044 (18)	0.0063 (17)	0.0063 (18)
C103	0.028 (2)	0.025 (2)	0.041 (2)	0.0015 (16)	0.0029 (17)	−0.0020 (18)
C104	0.0235 (19)	0.037 (2)	0.030 (2)	−0.0004 (17)	0.0044 (16)	−0.0028 (17)
C105	0.0235 (19)	0.027 (2)	0.038 (2)	0.0030 (16)	0.0074 (17)	0.0069 (17)
C106	0.025 (2)	0.037 (2)	0.040 (2)	0.0011 (17)	0.0017 (17)	−0.0086 (19)
C110	0.034 (2)	0.031 (2)	0.035 (2)	0.0038 (17)	0.0062 (17)	0.0032 (18)
C111	0.040 (2)	0.044 (3)	0.046 (3)	0.007 (2)	0.006 (2)	0.008 (2)
C112	0.055 (3)	0.047 (3)	0.089 (4)	0.020 (2)	0.023 (3)	0.022 (3)
C113	0.026 (2)	0.048 (3)	0.033 (2)	−0.0027 (18)	−0.0020 (17)	0.0011 (19)
C114	0.029 (2)	0.089 (4)	0.033 (2)	−0.004 (2)	−0.0002 (18)	0.004 (2)
C115	0.032 (2)	0.107 (5)	0.041 (3)	−0.012 (3)	−0.005 (2)	0.006 (3)
C116	0.029 (2)	0.041 (2)	0.044 (3)	−0.0063 (18)	0.0038 (18)	0.001 (2)
C117	0.029 (2)	0.060 (3)	0.048 (3)	−0.005 (2)	0.003 (2)	−0.009 (2)
C118	0.047 (3)	0.078 (4)	0.072 (4)	−0.006 (3)	−0.013 (3)	0.008 (3)
C119	0.037 (2)	0.034 (2)	0.041 (2)	0.0024 (18)	0.0041 (19)	−0.0083 (19)
C120	0.040 (2)	0.038 (2)	0.045 (3)	0.008 (2)	0.007 (2)	0.001 (2)
C121	0.046 (3)	0.070 (4)	0.062 (3)	0.022 (3)	0.011 (2)	0.013 (3)
C122	0.031 (2)	0.048 (3)	0.054 (3)	0.002 (2)	0.005 (2)	0.002 (2)
C123	0.038 (3)	0.078 (4)	0.069 (3)	0.006 (3)	0.012 (2)	−0.002 (3)
C124	0.032 (3)	0.059 (3)	0.091 (4)	−0.002 (2)	−0.005 (3)	−0.007 (3)
C125	0.040 (3)	0.055 (3)	0.069 (3)	0.007 (2)	0.001 (2)	0.004 (3)
C126	0.036 (2)	0.041 (3)	0.054 (3)	0.008 (2)	−0.003 (2)	0.000 (2)
C127	0.060 (3)	0.039 (3)	0.054 (3)	0.016 (2)	0.001 (2)	0.002 (2)
C128	0.044 (3)	0.065 (3)	0.077 (4)	0.023 (3)	0.012 (3)	0.013 (3)
C129	0.049 (3)	0.057 (3)	0.093 (4)	0.011 (3)	−0.023 (3)	−0.018 (3)
C130	0.023 (2)	0.038 (2)	0.049 (3)	0.0046 (18)	0.0018 (18)	−0.002 (2)
C131	0.029 (2)	0.088 (4)	0.062 (3)	0.009 (3)	−0.005 (2)	0.002 (3)
C132	0.033 (2)	0.048 (3)	0.073 (3)	0.004 (2)	0.015 (2)	−0.013 (2)
C133	0.025 (2)	0.046 (3)	0.090 (4)	0.000 (2)	0.011 (2)	−0.005 (3)
C134	0.024 (2)	0.040 (3)	0.082 (4)	0.0045 (19)	0.005 (2)	0.004 (2)
C135	0.028 (4)	0.046 (4)	0.069 (5)	0.012 (3)	0.005 (3)	0.023 (4)
C136	0.036 (4)	0.068 (5)	0.074 (5)	0.022 (4)	0.007 (4)	−0.005 (4)
C137	0.025 (4)	0.046 (4)	0.084 (6)	0.005 (3)	−0.005 (4)	0.013 (4)
C335	0.040 (7)	0.106 (10)	0.140 (11)	0.024 (7)	−0.005 (7)	0.024 (9)
C336	0.030 (6)	0.132 (10)	0.131 (10)	0.030 (7)	0.002 (6)	0.012 (9)
C337	0.044 (7)	0.160 (13)	0.132 (12)	0.038 (8)	−0.018 (8)	−0.006 (11)
O6	0.0312 (15)	0.0400 (17)	0.0535 (19)	0.0062 (13)	0.0019 (13)	0.0040 (14)
C107	0.158 (15)	0.108 (12)	0.104 (11)	0.000 (11)	−0.074 (10)	0.035 (10)
C108	0.182 (13)	0.114 (11)	0.112 (9)	−0.012 (10)	−0.085 (9)	0.024 (9)
C109	0.187 (14)	0.132 (12)	0.116 (10)	−0.014 (11)	−0.079 (10)	0.012 (10)
C207	0.021 (5)	0.040 (6)	0.060 (7)	0.016 (4)	−0.003 (5)	0.006 (5)
C208	0.043 (5)	0.058 (6)	0.073 (6)	0.010 (5)	−0.031 (4)	0.007 (5)
C209	0.068 (6)	0.054 (6)	0.067 (6)	0.006 (5)	−0.033 (5)	0.006 (5)
C138	0.066 (4)	0.054 (3)	0.054 (4)	0.023 (3)	0.025 (3)	0.003 (3)
C139	0.109 (5)	0.089 (5)	0.106 (5)	0.033 (4)	0.031 (4)	−0.025 (4)
C140	0.079 (4)	0.110 (5)	0.078 (4)	0.057 (4)	0.038 (4)	0.033 (4)
C141	0.086 (5)	0.109 (6)	0.071 (5)	0.053 (5)	0.050 (4)	0.035 (5)
C338	0.058 (8)	0.047 (8)	0.034 (8)	0.024 (8)	0.016 (7)	0.001 (7)
C339	0.054 (9)	0.045 (9)	0.020 (8)	0.028 (8)	0.010 (8)	−0.006 (8)
C340	0.053 (11)	0.045 (10)	0.030 (10)	0.029 (9)	0.005 (9)	−0.005 (9)
C341	0.051 (13)	0.044 (12)	0.038 (12)	0.026 (11)	0.007 (11)	−0.001 (11)
C67	0.024 (5)	0.040 (7)	0.041 (6)	−0.004 (5)	0.008 (4)	0.004 (5)
C68	0.052 (5)	0.048 (5)	0.060 (5)	−0.014 (4)	0.013 (4)	−0.001 (4)
C69	0.067 (5)	0.046 (5)	0.055 (5)	−0.008 (4)	0.004 (4)	−0.016 (4)
C70	0.065 (6)	0.052 (5)	0.079 (7)	0.012 (5)	−0.017 (5)	−0.013 (5)
C267	0.082 (13)	0.040 (10)	0.073 (11)	0.002 (10)	−0.006 (10)	−0.029 (9)
C268	0.098 (10)	0.051 (8)	0.092 (10)	−0.002 (7)	−0.004 (8)	−0.030 (7)
C269	0.116 (10)	0.044 (7)	0.096 (9)	−0.003 (7)	0.016 (8)	−0.025 (6)
C270	0.122 (12)	0.037 (7)	0.094 (10)	0.006 (8)	−0.007 (9)	−0.014 (8)
C36	0.041 (5)	0.035 (5)	0.052 (4)	−0.012 (4)	0.009 (3)	0.012 (3)
C37	0.078 (5)	0.034 (4)	0.051 (5)	0.004 (4)	0.013 (4)	0.005 (4)
C38	0.079 (5)	0.044 (4)	0.058 (4)	−0.001 (3)	0.005 (4)	0.008 (3)
C236	0.033 (9)	0.031 (9)	0.067 (9)	−0.011 (9)	0.013 (8)	0.015 (8)
C237	0.072 (9)	0.037 (8)	0.046 (9)	−0.002 (7)	0.017 (8)	0.006 (7)
C238	0.072 (9)	0.042 (9)	0.072 (9)	−0.005 (8)	−0.001 (8)	−0.004 (8)

### Geometric parameters (Å, °)

**Table:**

Cl1—C71	1.738 (7)	C89—C90	1.404 (5)
Cl2—C71	1.742 (6)	C89—H891	0.967
O1—C31	1.396 (5)	C90—C91	1.523 (5)
O1—C36	1.435 (8)	C90—C104	1.400 (5)
O1—C31	1.396 (5)	C91—C92	1.514 (5)
O1—C236	1.459 (18)	C91—H911	1.015
O2—C32	1.395 (5)	C91—H912	0.992
O2—C39	1.446 (5)	C92—C93	1.389 (5)
O3—C33	1.391 (5)	C92—C105	1.400 (5)
O3—C42	1.426 (5)	C93—C94	1.391 (5)
O4—C34	1.400 (5)	C93—H931	0.978
O4—C45	1.439 (5)	C94—C95	1.391 (6)
O5—C35	1.389 (5)	C94—C134	1.539 (5)
O5—C48	1.439 (5)	C95—C96	1.398 (5)
O7—C103	1.398 (4)	C95—H951	0.974
O7—C110	1.443 (4)	C96—C97	1.519 (5)
O8—C104	1.391 (4)	C96—C105	1.388 (5)
O8—C113	1.448 (4)	C97—C98	1.521 (5)
O9—C105	1.399 (4)	C97—H972	1.003
O9—C116	1.435 (5)	C97—H971	1.004
O10—C106	1.392 (4)	C98—C99	1.390 (5)
O10—C119	1.448 (5)	C98—C106	1.398 (6)
C1—C2	1.520 (5)	C99—C100	1.393 (6)
C1—C30	1.396 (6)	C99—H991	0.991
C1—C35	1.400 (5)	C100—C101	1.387 (6)
C2—C3	1.529 (5)	C100—C138	1.529 (11)
C2—H21	1.016	C100—C101	1.387 (6)
C2—H22	1.007	C100—C338	1.56 (5)
C3—C4	1.395 (5)	C101—H1011	0.987
C3—C31	1.393 (5)	C102—O6	1.393 (4)
C4—C5	1.394 (5)	C110—C111	1.506 (5)
C4—H41	0.970	C110—H1101	0.999
C5—C6	1.389 (5)	C110—H1102	1.024
C5—C51	1.536 (6)	C111—C112	1.519 (6)
C6—C7	1.388 (5)	C111—H1112	1.010
C6—H61	0.977	C111—H1111	1.005
C7—C8	1.516 (5)	C112—H1123	0.989
C7—C31	1.402 (5)	C112—H1122	0.984
C8—C9	1.524 (5)	C112—H1121	0.984
C8—H81	1.005	C113—C114	1.506 (5)
C8—H82	1.007	C113—H1131	1.007
C9—C10	1.394 (6)	C113—H1132	1.015
C9—C32	1.402 (5)	C114—C115	1.515 (6)
C10—C11	1.398 (6)	C114—H1142	1.016
C10—H101	0.984	C114—H1141	1.002
C11—C12	1.399 (6)	C115—H1153	0.956
C11—C55	1.527 (6)	C115—H1152	0.992
C12—C13	1.398 (6)	C115—H1151	1.000
C12—H121	0.990	C116—C117	1.505 (5)
C13—C14	1.521 (5)	C116—H1161	1.041
C13—C32	1.387 (5)	C116—H1162	1.002
C14—C15	1.518 (5)	C117—C118	1.516 (6)
C14—H141	1.006	C117—H1171	1.001
C14—H142	1.009	C117—H1172	1.017
C15—C16	1.392 (6)	C118—H1182	0.985
C15—C33	1.401 (6)	C118—H1181	0.980
C16—C17	1.392 (6)	C118—H1183	0.979
C16—H161	0.988	C119—C120	1.506 (5)
C17—C18	1.391 (6)	C119—H1192	1.018
C17—C59	1.531 (6)	C119—H1191	1.008
C18—C19	1.392 (6)	C120—C121	1.500 (6)
C18—H181	0.983	C120—H1201	1.023
C19—C20	1.517 (6)	C120—H1202	1.002
C19—C33	1.392 (5)	C121—H1212	0.981
C20—C21	1.536 (6)	C121—H1211	0.988
C20—H202	1.003	C121—H1213	0.976
C20—H201	1.012	C122—C123	1.541 (6)
C21—C22	1.391 (6)	C122—C124	1.522 (6)
C21—C34	1.393 (6)	C122—C125	1.525 (6)
C22—C23	1.395 (6)	C123—H1232	0.979
C22—H221	0.987	C123—H1231	0.990
C23—C24	1.399 (6)	C123—H1233	0.997
C23—C63	1.533 (6)	C124—H1241	0.970
C24—C25	1.404 (6)	C124—H1243	0.975
C24—H241	0.993	C124—H1242	1.001
C25—C26	1.503 (6)	C125—H1251	0.973
C25—C34	1.386 (6)	C125—H1253	0.982
C26—C27	1.525 (5)	C125—H1252	0.991
C26—H261	1.007	C126—C127	1.533 (6)
C26—H262	0.991	C126—C128	1.532 (6)
C27—C28	1.378 (6)	C126—C129	1.544 (6)
C27—C35	1.397 (6)	C127—H1271	0.988
C28—C29	1.398 (6)	C127—H1272	0.995
C28—H281	0.990	C127—H1273	0.988
C29—C30	1.393 (6)	C128—H1282	0.985
C29—C267	1.52 (3)	C128—H1281	0.995
C29—C30	1.393 (6)	C128—H1283	0.986
C29—C67	1.537 (18)	C129—H1292	0.976
C30—H301	0.971	C129—H1291	0.977
C39—C40	1.485 (6)	C129—H1293	0.987
C39—H392	1.025	C130—C131	1.521 (6)
C39—H391	1.006	C130—C132	1.533 (6)
C40—C41	1.521 (7)	C130—C133	1.525 (6)
C40—H401	1.014	C131—H1311	0.980
C40—H402	1.018	C131—H1313	0.991
C41—H413	0.985	C131—H1312	0.986
C41—H412	0.982	C132—H1321	0.987
C41—H411	1.002	C132—H1323	0.999
C42—C43	1.515 (7)	C132—H1322	0.976
C42—H422	1.040	C133—H1331	0.970
C42—H421	1.009	C133—H1332	0.994
C43—C44	1.430 (9)	C133—H1333	0.993
C43—H431	1.031	C134—C135	1.611 (8)
C43—H432	1.036	C134—C136	1.464 (8)
C44—H441	0.987	C134—C137	1.555 (8)
C44—H443	1.029	C134—C335	1.531 (12)
C44—H442	0.969	C134—C336	1.626 (12)
C45—C46	1.510 (7)	C134—C337	1.407 (13)
C45—H451	1.016	C135—H1352	0.955
C45—H452	1.014	C135—H1351	0.940
C46—C47	1.504 (9)	C135—H1353	0.957
C46—H462	1.010	C136—H1362	0.979
C46—H461	1.008	C136—H1361	0.968
C47—H471	0.983	C136—H1363	0.996
C47—H473	1.011	C137—H1371	0.978
C47—H472	0.993	C137—H1373	0.977
C48—C49	1.495 (6)	C137—H1372	0.982
C48—H481	1.018	C335—H3351	0.973
C48—H482	0.993	C335—H3353	0.971
C49—C50	1.511 (7)	C335—H3352	0.944
C49—H492	1.008	C336—H3361	0.953
C49—H491	1.005	C336—H3362	0.950
C50—H501	0.985	C336—H3363	1.003
C50—H502	1.003	C337—H3372	1.035
C50—H503	0.986	C337—H3371	0.866
C51—C52	1.527 (6)	C337—H3373	1.085
C51—C53	1.539 (6)	O6—C107	1.439 (15)
C51—C54	1.536 (6)	O6—C207	1.426 (11)
C52—H522	0.981	C107—C108	1.544 (16)
C52—H521	0.985	C107—H1072	1.037
C52—H523	0.996	C107—H1071	1.013
C53—H532	0.976	C108—C109	1.422 (15)
C53—H531	0.987	C108—H1081	0.980
C53—H533	0.983	C108—H1082	0.975
C54—H543	0.988	C109—H1093	0.942
C54—H542	1.002	C109—H1092	1.002
C54—H541	0.982	C109—H1091	0.979
C55—C56	1.515 (7)	C207—C208	1.532 (12)
C55—C57	1.511 (6)	C207—H2072	0.957
C55—C58	1.527 (7)	C207—H2071	1.002
C56—H563	0.984	C208—C209	1.500 (12)
C56—H562	0.988	C208—H2081	1.018
C56—H561	0.973	C208—H2082	0.983
C57—H571	0.977	C209—H2093	0.970
C57—H572	0.984	C209—H2092	0.958
C57—H573	0.982	C209—H2091	0.955
C58—H582	0.975	C138—C139	1.532 (9)
C58—H581	1.001	C138—C140	1.524 (9)
C58—H583	0.990	C138—C141	1.517 (9)
C59—C60	1.526 (7)	C139—H1393	0.983
C59—C61	1.523 (7)	C139—H1392	0.977
C59—C62	1.539 (6)	C139—H1391	0.986
C60—H602	1.000	C140—H1401	0.981
C60—H601	0.988	C140—H1402	0.995
C60—H603	1.002	C140—H1403	0.971
C61—H613	0.976	C141—H1412	0.967
C61—H612	0.968	C141—H1411	0.995
C61—H611	1.003	C141—H1413	0.989
C62—H623	0.975	C338—C339	1.53 (2)
C62—H622	0.991	C338—C340	1.53 (2)
C62—H621	0.993	C338—C341	1.53 (2)
C63—C64	1.544 (6)	C339—H3392	1.110
C63—C65	1.524 (6)	C339—H3391	1.129
C63—C66	1.529 (6)	C339—H3393	1.123
C64—H641	0.994	C340—H3403	0.997
C64—H642	0.984	C340—H3402	0.993
C64—H643	1.003	C340—H3401	0.879
C65—H652	0.997	C341—H3413	0.971
C65—H651	0.998	C341—H3412	0.926
C65—H653	0.977	C341—H3411	1.012
C66—H662	0.990	C67—C68	1.545 (11)
C66—H661	0.989	C67—C69	1.548 (12)
C66—H663	0.983	C67—C70	1.533 (12)
C71—H712	1.009	C68—H681	0.977
C71—H711	1.031	C68—H682	0.950
C72—C73	1.518 (5)	C68—H683	1.004
C72—C101	1.399 (5)	C69—H692	0.966
C72—C106	1.391 (5)	C69—H691	0.995
C73—C74	1.527 (5)	C69—H693	0.963
C73—H731	0.997	C70—H702	0.983
C73—H732	1.006	C70—H701	0.984
C74—C75	1.388 (5)	C70—H703	0.937
C74—C102	1.395 (5)	C267—C268	1.529 (17)
C75—C76	1.397 (6)	C267—C269	1.520 (17)
C75—H751	0.978	C267—C270	1.533 (17)
C76—C77	1.394 (6)	C268—H2683	0.919
C76—C122	1.531 (5)	C268—H2682	1.018
C77—C78	1.400 (5)	C268—H2681	1.018
C77—H771	0.962	C269—H2693	0.934
C78—C79	1.516 (5)	C269—H2692	0.976
C78—C102	1.385 (5)	C269—H2691	0.995
C79—C80	1.523 (5)	C270—H2701	0.942
C79—H791	0.993	C270—H2702	1.026
C79—H792	1.016	C270—H2703	0.968
C80—C81	1.388 (5)	C36—C37	1.494 (10)
C80—C103	1.396 (5)	C36—H362	0.992
C81—C82	1.389 (6)	C36—H361	1.002
C81—H811	0.968	C37—C38	1.506 (8)
C82—C83	1.382 (6)	C37—H372	1.006
C82—C126	1.533 (5)	C37—H371	0.996
C83—C84	1.399 (5)	C38—H381	0.982
C83—H831	0.964	C38—H382	0.969
C84—C85	1.526 (5)	C38—H383	0.983
C84—C103	1.404 (5)	C236—C237	1.509 (18)
C85—C86	1.527 (5)	C236—H2362	0.970
C85—H851	1.007	C236—H2361	1.024
C85—H852	1.019	C237—C238	1.512 (16)
C86—C87	1.407 (5)	C237—H2372	0.948
C86—C104	1.398 (5)	C237—H2371	0.940
C87—C88	1.387 (5)	C238—H2381	0.972
C87—H871	0.973	C238—H2382	0.955
C88—C89	1.384 (5)	C238—H2383	1.003
C88—C130	1.537 (5)		
			
C31—O1—C36	115.7 (4)	C98—C97—H972	108.5
C31—O1—C236	105.8 (9)	C96—C97—H971	109.5
C32—O2—C39	113.6 (3)	C98—C97—H971	108.4
C33—O3—C42	115.0 (3)	H972—C97—H971	108.1
C34—O4—C45	113.1 (3)	C97—C98—C99	121.0 (4)
C35—O5—C48	113.2 (3)	C97—C98—C106	120.6 (4)
C103—O7—C110	114.5 (3)	C99—C98—C106	118.5 (4)
C104—O8—C113	112.9 (3)	C98—C99—C100	122.4 (4)
C105—O9—C116	112.3 (3)	C98—C99—H991	118.0
C106—O10—C119	114.7 (3)	C100—C99—H991	119.6
C2—C1—C30	121.5 (4)	C99—C100—C101	117.1 (4)
C2—C1—C35	120.9 (4)	C99—C100—C138	121.3 (4)
C30—C1—C35	117.4 (4)	C101—C100—C138	121.7 (4)
C1—C2—C3	115.8 (3)	C99—C100—C101	117.1 (4)
C1—C2—H21	109.1	C99—C100—C338	122.4 (12)
C3—C2—H21	108.1	C101—C100—C338	120.2 (11)
C1—C2—H22	108.7	C72—C101—C100	122.8 (4)
C3—C2—H22	107.5	C72—C101—H1011	118.2
H21—C2—H22	107.4	C100—C101—H1011	119.0
C2—C3—C4	120.6 (4)	C74—C102—C78	121.6 (4)
C2—C3—C31	121.5 (4)	C74—C102—O6	119.7 (4)
C4—C3—C31	117.8 (4)	C78—C102—O6	118.6 (3)
C3—C4—C5	122.9 (4)	C84—C103—O7	119.6 (3)
C3—C4—H41	119.2	C84—C103—C80	121.4 (3)
C5—C4—H41	117.9	O7—C103—C80	118.7 (3)
C4—C5—C6	116.7 (4)	C90—C104—C86	121.3 (3)
C4—C5—C51	122.4 (4)	C90—C104—O8	118.0 (3)
C6—C5—C51	120.8 (4)	C86—C104—O8	120.7 (3)
C5—C6—C7	123.2 (4)	C92—C105—O9	118.5 (3)
C5—C6—H61	119.1	C92—C105—C96	121.6 (3)
C7—C6—H61	117.7	O9—C105—C96	119.9 (3)
C6—C7—C8	120.5 (4)	C98—C106—O10	118.1 (4)
C6—C7—C31	117.8 (4)	C98—C106—C72	121.2 (4)
C8—C7—C31	121.6 (4)	O10—C106—C72	120.6 (4)
C7—C8—C9	116.3 (3)	O7—C110—C111	108.2 (3)
C7—C8—H81	108.2	O7—C110—H1101	109.2
C9—C8—H81	107.4	C111—C110—H1101	111.0
C7—C8—H82	108.3	O7—C110—H1102	109.2
C9—C8—H82	107.5	C111—C110—H1102	111.0
H81—C8—H82	108.9	H1101—C110—H1102	108.2
C8—C9—C10	120.1 (4)	C110—C111—C112	109.9 (4)
C8—C9—C32	122.2 (4)	C110—C111—H1112	107.7
C10—C9—C32	117.7 (4)	C112—C111—H1112	110.9
C9—C10—C11	123.2 (4)	C110—C111—H1111	109.2
C9—C10—H101	118.5	C112—C111—H1111	110.7
C11—C10—H101	118.3	H1112—C111—H1111	108.3
C10—C11—C12	116.2 (4)	C111—C112—H1123	110.9
C10—C11—C55	121.6 (4)	C111—C112—H1122	110.5
C12—C11—C55	122.2 (4)	H1123—C112—H1122	107.0
C11—C12—C13	122.9 (4)	C111—C112—H1121	112.1
C11—C12—H121	119.2	H1123—C112—H1121	108.8
C13—C12—H121	117.9	H1122—C112—H1121	107.4
C12—C13—C14	120.3 (4)	O8—C113—C114	109.1 (3)
C12—C13—C32	118.0 (4)	O8—C113—H1131	110.4
C14—C13—C32	121.7 (4)	C114—C113—H1131	111.2
C13—C14—C15	114.8 (3)	O8—C113—H1132	109.7
C13—C14—H141	107.7	C114—C113—H1132	108.5
C15—C14—H141	108.3	H1131—C113—H1132	107.8
C13—C14—H142	108.2	C113—C114—C115	111.6 (4)
C15—C14—H142	109.3	C113—C114—H1142	109.0
H141—C14—H142	108.4	C115—C114—H1142	109.0
C14—C15—C16	119.9 (4)	C113—C114—H1141	108.5
C14—C15—C33	121.1 (4)	C115—C114—H1141	110.5
C16—C15—C33	119.1 (4)	H1142—C114—H1141	108.1
C15—C16—C17	122.0 (4)	C114—C115—H1153	111.1
C15—C16—H161	118.7	C114—C115—H1152	110.8
C17—C16—H161	119.3	H1153—C115—H1152	109.2
C16—C17—C18	117.0 (4)	C114—C115—H1151	108.6
C16—C17—C59	122.8 (4)	H1153—C115—H1151	108.8
C18—C17—C59	120.2 (4)	H1152—C115—H1151	108.4
C17—C18—C19	123.3 (4)	O9—C116—C117	108.9 (3)
C17—C18—H181	118.8	O9—C116—H1161	110.0
C19—C18—H181	118.0	C117—C116—H1161	110.9
C18—C19—C20	119.9 (4)	O9—C116—H1162	108.8
C18—C19—C33	118.0 (4)	C117—C116—H1162	109.5
C20—C19—C33	122.1 (4)	H1161—C116—H1162	108.8
C19—C20—C21	112.6 (3)	C116—C117—C118	113.4 (4)
C19—C20—H202	109.3	C116—C117—H1171	108.6
C21—C20—H202	108.1	C118—C117—H1171	108.2
C19—C20—H201	109.3	C116—C117—H1172	110.3
C21—C20—H201	109.4	C118—C117—H1172	109.1
H202—C20—H201	108.0	H1171—C117—H1172	107.0
C20—C21—C22	120.8 (4)	C117—C118—H1182	110.7
C20—C21—C34	121.5 (4)	C117—C118—H1181	109.9
C22—C21—C34	117.8 (4)	H1182—C118—H1181	109.1
C21—C22—C23	123.3 (4)	C117—C118—H1183	109.2
C21—C22—H221	118.5	H1182—C118—H1183	109.5
C23—C22—H221	118.3	H1181—C118—H1183	108.4
C22—C23—C24	116.6 (4)	O10—C119—C120	108.9 (3)
C22—C23—C63	120.0 (4)	O10—C119—H1192	110.0
C24—C23—C63	123.3 (4)	C120—C119—H1192	110.1
C23—C24—C25	122.1 (4)	O10—C119—H1191	109.0
C23—C24—H241	119.4	C120—C119—H1191	110.3
C25—C24—H241	118.5	H1192—C119—H1191	108.5
C24—C25—C26	119.2 (4)	C119—C120—C121	114.0 (4)
C24—C25—C34	118.3 (4)	C119—C120—H1201	108.9
C26—C25—C34	122.5 (4)	C121—C120—H1201	108.2
C25—C26—C27	117.6 (3)	C119—C120—H1202	108.3
C25—C26—H261	107.6	C121—C120—H1202	110.2
C27—C26—H261	106.1	H1201—C120—H1202	107.1
C25—C26—H262	107.3	C120—C121—H1212	111.1
C27—C26—H262	108.9	C120—C121—H1211	109.4
H261—C26—H262	109.0	H1212—C121—H1211	108.4
C26—C27—C28	119.6 (4)	C120—C121—H1213	109.3
C26—C27—C35	121.7 (4)	H1212—C121—H1213	110.1
C28—C27—C35	118.6 (4)	H1211—C121—H1213	108.4
C27—C28—C29	122.8 (4)	C76—C122—C123	108.5 (4)
C27—C28—H281	118.6	C76—C122—C124	112.4 (4)
C29—C28—H281	118.6	C123—C122—C124	108.6 (4)
C28—C29—C30	116.7 (4)	C76—C122—C125	110.8 (4)
C28—C29—C267	121.4 (8)	C123—C122—C125	108.3 (4)
C30—C29—C267	121.8 (8)	C124—C122—C125	108.2 (4)
C28—C29—C30	116.7 (4)	C122—C123—H1232	110.0
C28—C29—C67	122.5 (5)	C122—C123—H1231	109.5
C30—C29—C67	120.9 (5)	H1232—C123—H1231	109.0
C1—C30—C29	123.1 (4)	C122—C123—H1233	110.5
C1—C30—H301	118.5	H1232—C123—H1233	109.9
C29—C30—H301	118.3	H1231—C123—H1233	107.9
C7—C31—O1	118.0 (3)	C122—C124—H1241	109.5
C7—C31—C3	121.5 (4)	C122—C124—H1243	110.0
O1—C31—C3	120.5 (3)	H1241—C124—H1243	110.7
C9—C32—O2	118.9 (3)	C122—C124—H1242	109.3
C9—C32—C13	121.6 (4)	H1241—C124—H1242	108.4
O2—C32—C13	119.5 (4)	H1243—C124—H1242	108.9
C15—C33—C19	120.7 (4)	C122—C125—H1251	110.0
C15—C33—O3	119.2 (4)	C122—C125—H1253	108.8
C19—C33—O3	119.8 (4)	H1251—C125—H1253	108.4
O4—C34—C21	119.3 (4)	C122—C125—H1252	110.7
O4—C34—C25	118.9 (4)	H1251—C125—H1252	109.2
C21—C34—C25	121.7 (4)	H1253—C125—H1252	109.6
C1—C35—C27	121.4 (4)	C82—C126—C127	109.2 (4)
C1—C35—O5	120.0 (4)	C82—C126—C128	112.5 (4)
C27—C35—O5	118.5 (4)	C127—C126—C128	108.0 (4)
O2—C39—C40	114.3 (4)	C82—C126—C129	108.8 (4)
O2—C39—H392	109.3	C127—C126—C129	109.0 (4)
C40—C39—H392	109.0	C128—C126—C129	109.2 (4)
O2—C39—H391	106.7	C126—C127—H1271	110.8
C40—C39—H391	108.3	C126—C127—H1272	109.3
H392—C39—H391	109.0	H1271—C127—H1272	108.6
C39—C40—C41	113.8 (5)	C126—C127—H1273	111.0
C39—C40—H401	107.6	H1271—C127—H1273	109.3
C41—C40—H401	108.9	H1272—C127—H1273	107.8
C39—C40—H402	109.2	C126—C128—H1282	110.2
C41—C40—H402	108.5	C126—C128—H1281	110.2
H401—C40—H402	108.7	H1282—C128—H1281	109.7
C40—C41—H413	111.0	C126—C128—H1283	109.9
C40—C41—H412	110.7	H1282—C128—H1283	107.6
H413—C41—H412	110.1	H1281—C128—H1283	109.2
C40—C41—H411	108.5	C126—C129—H1292	109.5
H413—C41—H411	108.6	C126—C129—H1291	109.0
H412—C41—H411	107.8	H1292—C129—H1291	108.6
O3—C42—C43	109.5 (4)	C126—C129—H1293	111.0
O3—C42—H422	107.0	H1292—C129—H1293	109.4
C43—C42—H422	111.6	H1291—C129—H1293	109.3
O3—C42—H421	110.6	C88—C130—C131	108.9 (3)
C43—C42—H421	110.5	C88—C130—C132	109.4 (3)
H422—C42—H421	107.6	C131—C130—C132	109.4 (4)
C42—C43—C44	116.9 (6)	C88—C130—C133	112.8 (3)
C42—C43—H431	109.6	C131—C130—C133	109.0 (4)
C44—C43—H431	106.0	C132—C130—C133	107.1 (4)
C42—C43—H432	109.9	C130—C131—H1311	111.8
C44—C43—H432	105.9	C130—C131—H1313	109.8
H431—C43—H432	108.1	H1311—C131—H1313	107.6
C43—C44—H441	110.8	C130—C131—H1312	109.6
C43—C44—H443	105.6	H1311—C131—H1312	108.7
H441—C44—H443	108.1	H1313—C131—H1312	109.3
C43—C44—H442	109.1	C130—C132—H1321	111.4
H441—C44—H442	112.7	C130—C132—H1323	110.7
H443—C44—H442	110.2	H1321—C132—H1323	107.7
O4—C45—C46	108.8 (4)	C130—C132—H1322	109.8
O4—C45—H451	111.5	H1321—C132—H1322	107.9
C46—C45—H451	110.2	H1323—C132—H1322	109.3
O4—C45—H452	108.7	C130—C133—H1331	111.1
C46—C45—H452	110.0	C130—C133—H1332	111.3
H451—C45—H452	107.7	H1331—C133—H1332	108.6
C45—C46—C47	111.8 (5)	C130—C133—H1333	109.5
C45—C46—H462	110.5	H1331—C133—H1333	108.4
C47—C46—H462	108.7	H1332—C133—H1333	107.8
C45—C46—H461	108.8	C94—C134—C135	107.4 (4)
C47—C46—H461	108.8	C94—C134—C136	117.2 (5)
H462—C46—H461	108.1	C135—C134—C136	107.6 (6)
C46—C47—H471	110.6	C94—C134—C137	110.0 (4)
C46—C47—H473	109.2	C135—C134—C137	102.6 (5)
H471—C47—H473	108.9	C136—C134—C137	110.9 (6)
C46—C47—H472	110.4	C94—C134—C335	106.3 (6)
H471—C47—H472	109.4	C94—C134—C336	104.2 (6)
H473—C47—H472	108.3	C335—C134—C336	96.3 (9)
O5—C48—C49	109.3 (4)	C94—C134—C337	115.0 (7)
O5—C48—H481	109.5	C335—C134—C337	123.9 (11)
C49—C48—H481	109.4	C336—C134—C337	107.8 (10)
O5—C48—H482	111.4	C134—C135—H1352	110.9
C49—C48—H482	109.7	C134—C135—H1351	109.9
H481—C48—H482	107.5	H1352—C135—H1351	108.2
C48—C49—C50	109.6 (4)	C134—C135—H1353	109.9
C48—C49—H492	110.0	H1352—C135—H1353	108.5
C50—C49—H492	110.2	H1351—C135—H1353	109.4
C48—C49—H491	108.7	C134—C136—H1362	111.2
C50—C49—H491	109.8	C134—C136—H1361	110.4
H492—C49—H491	108.6	H1362—C136—H1361	110.3
C49—C50—H501	110.3	C134—C136—H1363	109.2
C49—C50—H502	109.6	H1362—C136—H1363	107.1
H501—C50—H502	107.8	H1361—C136—H1363	108.5
C49—C50—H503	109.7	C134—C137—H1371	110.6
H501—C50—H503	110.5	C134—C137—H1373	111.3
H502—C50—H503	109.0	H1371—C137—H1373	110.5
C5—C51—C52	112.4 (3)	C134—C137—H1372	107.9
C5—C51—C53	108.7 (4)	H1371—C137—H1372	107.7
C52—C51—C53	107.5 (4)	H1373—C137—H1372	108.7
C5—C51—C54	109.9 (4)	C134—C335—H3351	109.7
C52—C51—C54	108.9 (4)	C134—C335—H3353	108.0
C53—C51—C54	109.4 (4)	H3351—C335—H3353	108.4
C51—C52—H522	110.5	C134—C335—H3352	110.5
C51—C52—H521	109.0	H3351—C335—H3352	110.1
H522—C52—H521	109.0	H3353—C335—H3352	110.1
C51—C52—H523	110.4	C134—C336—H3361	111.6
H522—C52—H523	108.7	C134—C336—H3362	111.2
H521—C52—H523	109.2	H3361—C336—H3362	110.4
C51—C53—H532	110.4	C134—C336—H3363	108.6
C51—C53—H531	110.2	H3361—C336—H3363	107.6
H532—C53—H531	108.1	H3362—C336—H3363	107.2
C51—C53—H533	110.0	C134—C337—H3372	108.6
H532—C53—H533	109.6	C134—C337—H3371	120.5
H531—C53—H533	108.5	H3372—C337—H3371	113.4
C51—C54—H543	109.9	C134—C337—H3373	105.0
C51—C54—H542	110.0	H3372—C337—H3373	97.7
H543—C54—H542	108.4	H3371—C337—H3373	108.7
C51—C54—H541	109.6	C102—O6—C107	113.0 (9)
H543—C54—H541	109.7	C102—O6—C207	114.2 (5)
H542—C54—H541	109.2	O6—C107—C108	112.6 (15)
C11—C55—C56	108.1 (4)	O6—C107—H1072	107.3
C11—C55—C57	112.8 (4)	C108—C107—H1072	110.9
C56—C55—C57	110.5 (5)	O6—C107—H1071	110.6
C11—C55—C58	111.4 (4)	C108—C107—H1071	111.9
C56—C55—C58	107.3 (5)	H1072—C107—H1071	103.1
C57—C55—C58	106.6 (4)	C107—C108—C109	99.5 (17)
C55—C56—H563	109.8	C107—C108—H1081	109.8
C55—C56—H562	107.7	C109—C108—H1081	115.8
H563—C56—H562	108.9	C107—C108—H1082	109.7
C55—C56—H561	109.8	C109—C108—H1082	110.6
H563—C56—H561	110.9	H1081—C108—H1082	110.9
H562—C56—H561	109.7	C108—C109—H1093	107.2
C55—C57—H571	111.0	C108—C109—H1092	108.1
C55—C57—H572	109.7	H1093—C109—H1092	110.0
H571—C57—H572	108.2	C108—C109—H1091	112.2
C55—C57—H573	111.6	H1093—C109—H1091	112.2
H571—C57—H573	108.9	H1092—C109—H1091	107.1
H572—C57—H573	107.3	O6—C207—C208	104.1 (8)
C55—C58—H582	109.5	O6—C207—H2072	112.4
C55—C58—H581	109.4	C208—C207—H2072	111.3
H582—C58—H581	110.6	O6—C207—H2071	111.9
C55—C58—H583	109.7	C208—C207—H2071	106.6
H582—C58—H583	109.6	H2072—C207—H2071	110.3
H581—C58—H583	108.0	C207—C208—C209	110.5 (10)
C17—C59—C60	112.1 (4)	C207—C208—H2081	112.6
C17—C59—C61	109.7 (5)	C209—C208—H2081	106.4
C60—C59—C61	108.5 (5)	C207—C208—H2082	110.6
C17—C59—C62	109.2 (4)	C209—C208—H2082	111.2
C60—C59—C62	106.7 (5)	H2081—C208—H2082	105.3
C61—C59—C62	110.5 (4)	C208—C209—H2093	110.0
C59—C60—H602	108.4	C208—C209—H2092	109.9
C59—C60—H601	111.8	H2093—C209—H2092	109.6
H602—C60—H601	108.8	C208—C209—H2091	109.0
C59—C60—H603	111.1	H2093—C209—H2091	110.0
H602—C60—H603	108.1	H2092—C209—H2091	108.4
H601—C60—H603	108.6	C100—C138—C139	107.9 (6)
C59—C61—H613	109.6	C100—C138—C140	110.2 (6)
C59—C61—H612	110.7	C139—C138—C140	108.9 (7)
H613—C61—H612	110.8	C100—C138—C141	112.7 (6)
C59—C61—H611	108.3	C139—C138—C141	108.7 (8)
H613—C61—H611	108.8	C140—C138—C141	108.5 (7)
H612—C61—H611	108.5	C138—C139—H1393	108.5
C59—C62—H623	110.1	C138—C139—H1392	109.7
C59—C62—H622	110.0	H1393—C139—H1392	109.0
H623—C62—H622	110.0	C138—C139—H1391	109.7
C59—C62—H621	111.0	H1393—C139—H1391	109.4
H623—C62—H621	107.9	H1392—C139—H1391	110.5
H622—C62—H621	107.8	C138—C140—H1401	110.2
C23—C63—C64	110.4 (3)	C138—C140—H1402	108.6
C23—C63—C65	108.1 (4)	H1401—C140—H1402	108.7
C64—C63—C65	108.9 (4)	C138—C140—H1403	110.4
C23—C63—C66	112.7 (4)	H1401—C140—H1403	108.9
C64—C63—C66	108.1 (4)	H1402—C140—H1403	110.0
C65—C63—C66	108.6 (4)	C138—C141—H1412	111.3
C63—C64—H641	108.9	C138—C141—H1411	109.1
C63—C64—H642	110.2	H1412—C141—H1411	108.3
H641—C64—H642	108.7	C138—C141—H1413	110.4
C63—C64—H643	109.9	H1412—C141—H1413	108.8
H641—C64—H643	109.0	H1411—C141—H1413	108.8
H642—C64—H643	110.2	C100—C338—C339	110 (2)
C63—C65—H652	109.1	C100—C338—C340	111 (2)
C63—C65—H651	110.3	C339—C338—C340	107 (2)
H652—C65—H651	108.2	C100—C338—C341	108 (2)
C63—C65—H653	110.4	C339—C338—C341	109 (3)
H652—C65—H653	109.0	C340—C338—C341	111 (3)
H651—C65—H653	109.8	C338—C339—H3392	106.5
C63—C66—H662	109.8	C338—C339—H3391	105.7
C63—C66—H661	110.5	H3392—C339—H3391	112.6
H662—C66—H661	109.0	C338—C339—H3393	107.6
C63—C66—H663	109.7	H3392—C339—H3393	112.3
H662—C66—H663	108.7	H3391—C339—H3393	111.5
H661—C66—H663	109.3	C338—C340—H3403	106.4
Cl2—C71—Cl1	113.0 (4)	C338—C340—H3402	105.9
Cl2—C71—H712	109.2	H3403—C340—H3402	104.1
Cl1—C71—H712	110.4	C338—C340—H3401	114.0
Cl2—C71—H711	108.0	H3403—C340—H3401	112.6
Cl1—C71—H711	109.8	H3402—C340—H3401	113.1
H712—C71—H711	106.1	C338—C341—H3413	110.7
C73—C72—C101	119.5 (4)	C338—C341—H3412	112.5
C73—C72—C106	122.5 (4)	H3413—C341—H3412	111.6
C101—C72—C106	118.0 (4)	C338—C341—H3411	109.0
C72—C73—C74	113.7 (3)	H3413—C341—H3411	104.8
C72—C73—H731	109.0	H3412—C341—H3411	107.9
C74—C73—H731	108.8	C29—C67—C68	112.9 (9)
C72—C73—H732	108.2	C29—C67—C69	109.6 (9)
C74—C73—H732	109.6	C68—C67—C69	107.8 (10)
H731—C73—H732	107.4	C29—C67—C70	110.3 (9)
C73—C74—C75	121.7 (4)	C68—C67—C70	107.6 (10)
C73—C74—C102	120.4 (3)	C69—C67—C70	108.5 (9)
C75—C74—C102	117.9 (4)	C67—C68—H681	109.8
C74—C75—C76	122.8 (4)	C67—C68—H682	111.1
C74—C75—H751	118.7	H681—C68—H682	109.7
C76—C75—H751	118.4	C67—C68—H683	109.1
C75—C76—C77	116.7 (4)	H681—C68—H683	108.3
C75—C76—C122	119.9 (4)	H682—C68—H683	108.7
C77—C76—C122	123.4 (4)	C67—C69—H692	109.4
C76—C77—C78	122.4 (4)	C67—C69—H691	110.5
C76—C77—H771	119.4	H692—C69—H691	109.3
C78—C77—H771	118.2	C67—C69—H693	109.2
C77—C78—C79	120.2 (4)	H692—C69—H693	109.9
C77—C78—C102	118.0 (4)	H691—C69—H693	108.6
C79—C78—C102	121.7 (4)	C67—C70—H702	109.5
C78—C79—C80	117.5 (3)	C67—C70—H701	110.2
C78—C79—H791	108.5	H702—C70—H701	107.6
C80—C79—H791	108.6	C67—C70—H703	110.6
C78—C79—H792	106.8	H702—C70—H703	109.5
C80—C79—H792	107.2	H701—C70—H703	109.4
H791—C79—H792	107.9	C29—C267—C268	110.5 (16)
C79—C80—C81	119.7 (4)	C29—C267—C269	111.4 (16)
C79—C80—C103	122.3 (4)	C268—C267—C269	104.5 (17)
C81—C80—C103	117.9 (4)	C29—C267—C270	108.4 (15)
C80—C81—C82	123.3 (4)	C268—C267—C270	111.2 (18)
C80—C81—H811	117.5	C269—C267—C270	110.7 (17)
C82—C81—H811	119.2	C267—C268—H2683	114.4
C81—C82—C83	116.8 (4)	C267—C268—H2682	109.4
C81—C82—C126	120.1 (4)	H2683—C268—H2682	110.6
C83—C82—C126	123.1 (4)	C267—C268—H2681	108.0
C82—C83—C84	123.3 (4)	H2683—C268—H2681	110.1
C82—C83—H831	118.5	H2682—C268—H2681	103.7
C84—C83—H831	118.2	C267—C269—H2693	112.0
C83—C84—C85	121.0 (3)	C267—C269—H2692	106.3
C83—C84—C103	117.3 (4)	H2693—C269—H2692	111.2
C85—C84—C103	121.7 (3)	C267—C269—H2691	108.2
C84—C85—C86	115.9 (3)	H2693—C269—H2691	111.2
C84—C85—H851	108.3	H2692—C269—H2691	107.7
C86—C85—H851	107.5	C267—C270—H2701	113.4
C84—C85—H852	108.4	C267—C270—H2702	107.4
C86—C85—H852	109.1	H2701—C270—H2702	106.6
H851—C85—H852	107.3	C267—C270—H2703	109.9
C85—C86—C87	120.4 (3)	H2701—C270—H2703	112.5
C85—C86—C104	122.3 (3)	H2702—C270—H2703	106.6
C87—C86—C104	117.3 (3)	O1—C36—C37	108.0 (7)
C86—C87—C88	123.2 (4)	O1—C36—H362	110.7
C86—C87—H871	118.4	C37—C36—H362	109.7
C88—C87—H871	118.4	O1—C36—H361	111.3
C87—C88—C89	117.5 (3)	C37—C36—H361	110.2
C87—C88—C130	119.4 (3)	H362—C36—H361	106.9
C89—C88—C130	123.1 (4)	C36—C37—C38	112.6 (6)
C88—C89—C90	122.2 (4)	C36—C37—H372	109.3
C88—C89—H891	119.1	C38—C37—H372	110.4
C90—C89—H891	118.7	C36—C37—H371	109.2
C89—C90—C91	119.8 (3)	C38—C37—H371	109.0
C89—C90—C104	118.4 (3)	H372—C37—H371	106.2
C91—C90—C104	121.6 (3)	C37—C38—H381	111.9
C90—C91—C92	117.4 (3)	C37—C38—H382	110.3
C90—C91—H911	108.6	H381—C38—H382	108.4
C92—C91—H911	107.0	C37—C38—H383	110.5
C90—C91—H912	108.5	H381—C38—H383	107.4
C92—C91—H912	108.0	H382—C38—H383	108.2
H911—C91—H912	106.9	O1—C236—C237	114.4 (17)
C91—C92—C93	120.2 (3)	O1—C236—H2362	110.8
C91—C92—C105	121.8 (3)	C237—C236—H2362	112.2
C93—C92—C105	117.9 (3)	O1—C236—H2361	106.8
C92—C93—C94	122.7 (4)	C237—C236—H2361	105.9
C92—C93—H931	117.6	H2362—C236—H2361	106.1
C94—C93—H931	119.7	C236—C237—C238	109.8 (17)
C93—C94—C95	117.2 (3)	C236—C237—H2372	109.1
C93—C94—C134	121.1 (4)	C238—C237—H2372	105.5
C95—C94—C134	121.7 (4)	C236—C237—H2371	109.2
C94—C95—C96	122.4 (4)	C238—C237—H2371	109.1
C94—C95—H951	119.2	H2372—C237—H2371	113.9
C96—C95—H951	118.4	C237—C238—H2381	110.3
C95—C96—C97	119.9 (4)	C237—C238—H2382	113.9
C95—C96—C105	118.0 (4)	H2381—C238—H2382	109.2
C97—C96—C105	122.1 (3)	C237—C238—H2383	109.9
C96—C97—C98	112.6 (3)	H2381—C238—H2383	105.8
C96—C97—H972	109.6	H2382—C238—H2383	107.4

### Hydrogen-bond geometry (Å, °)

**Table:**

*D*—H···*A*	*D*—H	H···*A*	*D*···*A*	*D*—H···*A*
C71—H711···O7	1.03	2.36	3.363 (8)	163
C48—H482···O1	0.99	2.60	3.351 (5)	134
C110—H1101···O8	1.00	2.57	3.361 (5)	136
C14—H142···O2	1.01	2.39	2.874 (5)	109
C120—H1202···O9	1.00	2.57	3.371 (5)	136
C14—H142···O3	1.01	2.47	2.869 (5)	103
C73—H731···O6	1.00	2.42	2.862 (5)	106
C73—H731···O10	1.00	2.50	2.918 (5)	105
C85—H852···O7	1.02	2.42	2.900 (5)	108
C85—H852···O8	1.02	2.46	2.924 (5)	107
C97—H972···O9	1.00	2.43	2.896 (5)	107
C97—H972···O10	1.00	2.46	2.829 (5)	101
C38—H382···Cl1	0.97	2.92	3.795 (5)	151
